# Clinical symbiosis of hybrid nanoparticles and induced magnetic field on heat and mass transfer in multiple stenosed artery with erratic thrombosis

**DOI:** 10.1038/s41598-023-42795-7

**Published:** 2023-09-20

**Authors:** Azad Hussain, Muhammad Naveel Riaz Dar, Warda Khalid Cheema, Yanshuo Han, Rimsha Kanwal

**Affiliations:** 1 Department of Mathematics, University of Gujrat, Gujrat, 50700 Pakistan; 2 School of Life and Pharmaceutical Sciences, Dalian University of Technology, Dalian, China

**Keywords:** Biophysics, Energy science and technology, Mathematics and computing

## Abstract

This article scrutinizes blood circulation through an artery having magnetized hybrid nanoparticles (silver and gold) with multiple stenoses at the outer walls and erratic thrombus of different radii at the center. In the realm of biomedical innovation, magnetized hybrid nanoparticles emerge as a captivating frontier. These nanoparticles, amalgamating diverse materials, exhibit magnetic properties that engender novel prospects for targeted drug delivery, medical imaging enhancement, and therapeutic interventions. The study was carried out employing modern bio-fluid dynamics (BFD) software. In this iterative procedure, a second-order finite difference approach is used to solve the governing equations with 0.005 tolerance. The experiment is performed on a blood conduit with mild stenosis assumptions, and expressions of temperature, resistance impedance to flow, velocity, wall shear stress, and pressure gradient are generated by employing related boundary conditions. No one has ever attempted to acquire the remedial impact of an induced magnetic field and hybrid nanoparticles on the bloodstream in a tapering artery containing multiple stenoses on the outside walls and multi-thrombus at the center using 3-D bio-fluid simulation. Furthermore, the study's findings are unique, and these computational discoveries were not previously published by any researcher. The findings suggest that hybrid nanoparticles can be used as medication carriers to reduce the impact of thrombosis and stenosis-induced resistance to blood flow or coagulation-related factors.

## Introduction

According to research, the blood flow from and to the heart is a factor in more than 80% of deaths caused by heart disease^1^. A condition known as atherosclerosis occurs when fatty compounds build up on the arterial walls and harden them. Plaques are the common name for these fatty accumulations. Plaques that plug inside the arteries signal the presence of a dangerous illness known as arterial atherosclerosis. When a blood vessel or more structures like a tube narrows abnormally, it is referred to as stenosis in medicine. The slow accumulation of plaque, which is mostly made of cholesterol, fat, and cellular debris, on the inner walls of arteries is referred to as atherosclerosis. On the other hand, stenosis is the narrowing or constriction of an artery as a result of a variety of circumstances, such as the buildup of atherosclerotic plaque. In essence, stenosis can result from atherosclerosis when plaque formation narrows the artery lumen, obstructing blood flow and perhaps hurting health. There are essentially two tube-like structures in the spine. The narrowing of one or more areas within your spine is known as spinal stenosis. When your spine is compressed, the nerves and spinal cord that emerge from it have less room to travel. Young^2^ was the first to investigate stenosis; the author says that relatively minor growths cause moderate blockages. Mandal and Chakravarthy^3,4^ examine a non-Newtonian model of blood flow in a truncated overlapped confinement. Several biological systems do not have linear vessels, and some may have proclivity. Nadeem et al.^5^ used varying viscosity to theoretically describe the movement of nanotubes over an artery containing multiple stenoses. The effects of proclivity on the flow of blood via perfused arteries are investigated by many researchers^6–8^.

Another common kind of cardiovascular illness that can emerge as a consequence of stenosis worsening is thrombosis, which results from the establishment of a clot, which also barricades blood flow in the veins. Its progression can result in a variety of illnesses and ailments, including infarction, strokes, malignancy, and infections^9^. An arterial thrombus is a blood clot in the artery that can be deadly because it prevents blood from reaching vital organs. Several academics have recently focused on studying of bloodstream through arteries with thrombus in the center. Such blood clots (thrombus) grow more commonly in sick arteries with tapering walls. As a result, the artery with severe stenosis seems to be more likely to develop a thrombus. This is a life-threatening case that almost limits the flow of blood and it has catastrophic implications. In this case, catheter insertion improves flow once again. This thin, empty pipe can be inserted into such problematic arteries to improve the flow. Doffin et al.^10^ investigated the flow issue with both scenarios (stenosis and thrombus) both analytically and experimentally.

Bio-magnetic hydrodynamics is a novel field of fluid mechanics that studies the fluid dynamics of bio-fluids in the existence of magnetic fields. Several scientists are eager to learn more about the impact of magnetic flux on blood flow. The magnetic field significantly impacts bodily fluid dynamics, which has ramifications for biotechnology and medical equipment. As a result, the magnetic fields may affect the blood flow. Magnetic field applications associated with health disciplines are demonstrated in several contexts, including illness treatment. Nevertheless, Haik et al.^11^ discovered bio-magnetic fluid dynamics. Biological fluids are Ferro-fluids, which are magnetic fluids that do not carry electricity. Ferro-fluids are used in a variety of applications, including pharmaceuticals, and anti-tumor drug haulers. Magneto-therapy is a complementary medical treatment in which magnets are utilized to address pain and multi-health concerns in people with cardiovascular disease^12^. Researchers are interested in investigating basic bio-magnetic hydrodynamics flow problems because of the innumerable major applications in biomedical engineering and biological sciences, like the advancement of magnetic materials for cell parting, targeted therapies transfer employing magnetic nanoparticles as medication delivery transporters, magnetic help combat and cancer therapy instigating magnetic hypoglycemia, minimizing blood loss during surgical treatment, and incitement of occlusive disease. Another notable implementation of these liquids is in chemotherapy. The medicine is coated with magnetized nanoparticles and administered adjacent to the malignance in the treatment. The drug is immersed by the malignant tumor using an extravagant magnetic field focused around the tumor’s midpoint. This method diminishes the adverse effects of anticancer drugs. This approach has primarily been tested on tiny animals. Recently, several studies reported the use of such a technology for human therapy in cases when the tumor is close to the skin^13^.

Choi^14^ was the first who invented nano-fluids. These are manipulated colloids constituted of nanoparticles and the base fluid. The nanoparticles are composed of metals, oxides and carbon nanotubes. These nanoparticles contain thermal conductivity and have a magnitude greater than the base fluid. Also, the sizes of nanoparticles are lesser than 100 nm. Nanotechnology is a new discipline of research that involves the formation and advancement of different nanomaterials. The nanoparticles have broad applications in medical like as bio-medicines, because of how nanoparticles collaborate with matter^15^. The characteristics of nanoparticles like surface chemistry, shape, and size can be handled to increase their objectives in human circulatory systems. This article offers a moving through a perfused area to determine how hybridized could assist to improve blood flow. Nanoparticles may be utilized in the investigation, like mediators in optical, photoacoustic, in the delivery of drugs, as shippers capable to enhance cancer disclosure to a therapeutic assistant, developing treatment fallout by continuance circulation times, preserving transported drugs from deterioration and increasing tumor assimilation. The blood-mediated nanoparticles consignment is the expanded and latest area in the progression of therapeutics and diagnostics.

Hybrid nano-fluids are relatively novel forms of nanofluid that may be created by suspending distinct types (two or more) of nanoparticles in the base fluid, and hybrid (compound) nanoparticles in the base fluid. A hybrid constituent is a substance that blends the physical and biochemical properties of many substances at the same time and delivers the properties in a uniform phase. The physicochemical characteristics of synthesized hybrid nanostructures are exceptional since they do not present in the separate components. Ahmed and Nadeem^16^ studied the presence of minor stenosis plaques in the presence of several types of nanoparticles including copper (Cu), aluminum (Al_2_O_3_), and titanium dioxide (TiO_2_). Lubna et al.^17^ examined the impact of nano-fluids on a stenotic damaged artery. Zeeshan et al.^18^ investigated the effects of nanoparticle morphologies on mixed convective steady flow across a revolving disc. Copper nanoparticles of various sizes in disc, cylindrical, and spherical forms, as well as water as the base fluid, are studied for nanofluids. Nadeem^19^ investigated nanofluid flow in a curved duct with flexible walls. Mekheimer et al.^20,21^ investigated the effects of copper (Cu) nanoparticles on blood flow through a stenotic conduit in the presence of a magnetic field.

We looked over the existing research publications, and this data unambiguously reveals that no one has numerically and visually examined the blood flow within a vessel with multiple stenosis and multi-thrombosis with a varying radius in the presence of an induced magnetic field through three-dimensional computational simulation. Our study's goal is to fill up this significant research gap by venturing into unknown terrain and using the nonlinear Navier–Stokes equations to thoroughly examine the intricate fluid dynamics at work. To improve blood flow, our strategy also involves injecting hybrid nanoparticles into the body. By highlighting the possibility of creative approaches to improving blood circulation in situations characterized by numerous stenoses and multi-thrombosis, this research increases the practical value of our work. The precise results are further clarified by the inclusion of graphical representations, making this work helpful for theoretical insights as well as for guiding possible clinical applications and treatment approaches.

## Problem and mathematical model

**Figure 1:**
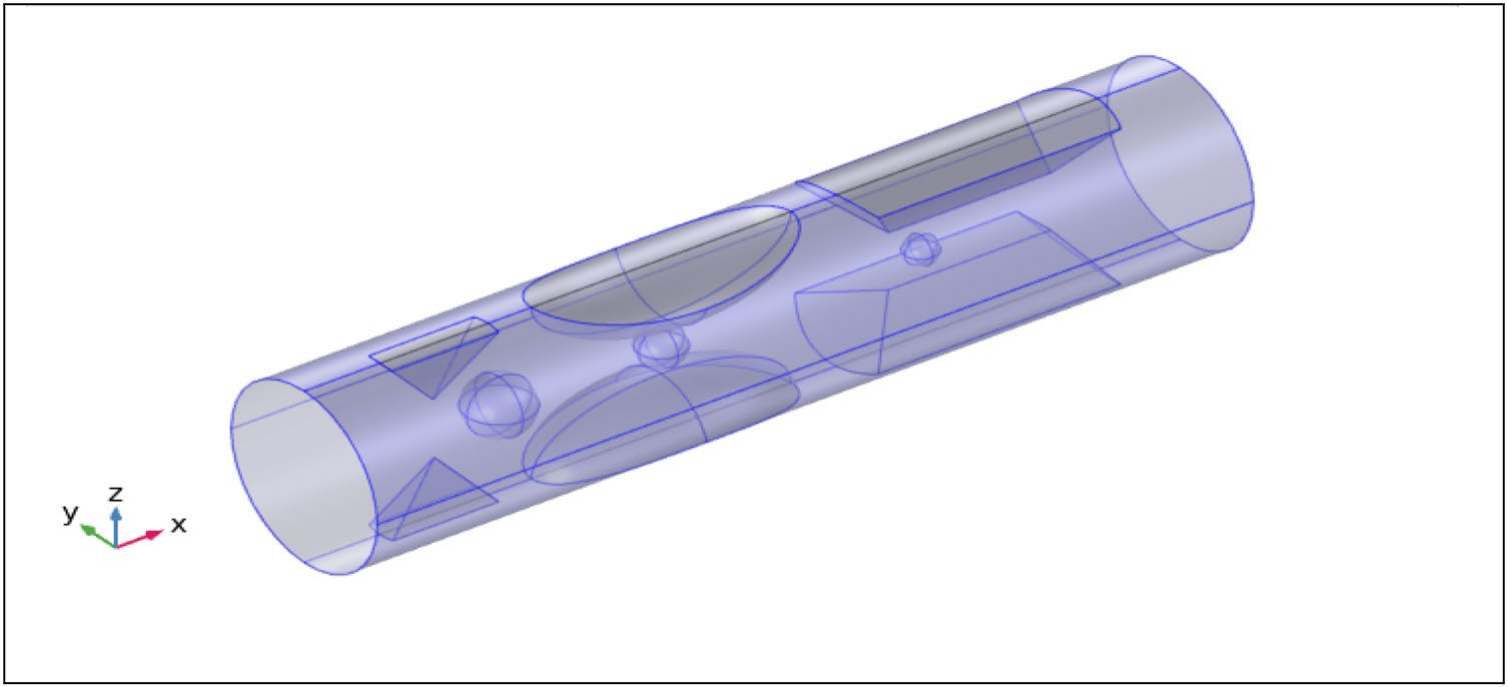
3-D geometry of multiple stenotic artery with multi-thrombosis.

Let us look into the computational simulation for the unsteady, incompressible, two-dimensional, and laminar blood flow across a circular artery of finite length L, containing hybrid nanoparticles (silver and gold) which are injected in the artery as a drug, passing through a thrombus (blood clot) of various radii developed at the center of the stenosed artery. A uniform magnetic field is applied to examine its effect on blood flow. The electrical conductivity of the blood is taken as 0.70 S/m and its relative permittivity is $5\times {10}^{3}$. The tube's wall has a relative permittivity and conductivity of $1.63\times {10}^{3}$ and 0.31 S/m respectively^22^. As illustrated in Fig. 1, it is preferable to work with the cylindrical coordinates (r, θ, z), where the z-axis is considered along the direction of the horizontal artery and θ and r the circumferential and radial directions, correspondingly. The flow is taken along the axial direction z and r is perpendicular to the flow.

The dimensions of time-dependent stenosis in the arterial wall $\widehat{\mathrm{R}}\left(\mathrm{x},\mathrm{t}\right)$ are specified as follows^23^:

1 $$\hat{R}\left( {x,t} \right) = \left\{ {\begin{array}{*{20}l} {\left[ {R - 2\mathop \delta \limits^{\prime } \left( {z - 2d} \right)} \right]\Omega \left( t \right)\quad d \le z \le d + l^{\prime}} \\ {\left[ {R - \mathop \delta \limits^{\prime } \left( {z - 2d - \frac{{l^{\prime}}}{2}} \right)} \right]\Omega \left( t \right)\quad d + l^{\prime} \le z \le 2l^{\prime}} \\ {} \\ {\left[ R \right]\Omega \left( t \right),\quad otherwise} \\ \end{array} } \right.$$

2 $$\hat{R}\left( {x,t} \right) = \left\{ {\begin{array}{*{20}l} {} \\ {\left[ {R - \mathop \delta \limits^{\prime } sin\left( {\pi \left( {\frac{{z - d}}{{l^{\prime} }}} \right)} \right)} \right]\Omega \left( t \right)\quad d \le z \le d + l^{\prime} } \\ {} \\ {\left[ R \right]\Omega \left( t \right),\quad otherwise} \\ \end{array} } \right.$$

3 $${\hat{\text{R}}}\left( {{\text{x}},{\text{t}}} \right) = \left\{ {\begin{array}{*{20}l} {\left[ {{\text{R}} - 2\mathop {{\delta }}\limits^{\prime } \left( {{\text{z}} - 2{\text{d}}} \right)} \right]\Omega \left( {\text{t}} \right)~\quad d \le z \le d + l^{\prime}} \\ {\left[ {{\text{R}} + \mathop {\frac{{{\delta }}}{5}}\limits^{\prime } \left( {\frac{{4l^{\prime}}}{5} - \frac{{11}}{5}{\text{d}}} \right)} \right]\Omega \left( {\text{t}} \right)~\quad d + l^{\prime} \le z \le 2l^{\prime}} \\ {\left[ {{\text{R}} + \mathop {\frac{{{\delta }}}{5}}\limits^{\prime } \left( {\frac{{4l^{\prime}}}{5} - \frac{{11}}{5}{\text{d}}} \right)} \right]\Omega \left( {\text{t}} \right)~\quad d + l^{\prime} \le z \le 2l^{\prime}} \\ {\left[ {\text{R}} \right]\Omega \left( {\text{t}} \right),~\quad otherwise} \\ \end{array} } \right.$$

The expression for the time-dependent parameter is $\Omega \left(\mathrm{t}\right)$ which was defined as:

$$\Omega \left(\mathrm{t}\right)=1-(\mathrm{cos}\omega t-1){e}^{-i\omega t}$$

where $\widehat{\mathrm{R}}\left(\mathrm{x},\mathrm{t}\right)$ is the radius of the restricted artery, R is the radius of the healthy artery in the non-restricted part, d is the position of stenosis, ${l}{^{\prime}}$ is the length of a stenosis, t denotes the passage of time, λ is a constant, ω is the angular frequency of imposed fluctuation, $\mathop \delta \limits^{\prime }$ is the severity or maximal height of the restricted area.

Correspondingly, the equations of an unsteady, viscous, and incompressible, hybrid nano-fluid along a stenotic artery having thrombosis and applying a magnetic field are given as^24^:

4 $$\rho \frac{\partial \mathbf{u}}{\partial t}+\rho \left(\mathbf{u}\cdot \nabla \right)\mathbf{u}=\nabla \cdot \left[-p\mathbf{I}]+\nabla [{\varvec{K}}\right]+{\varvec{F}},$$

where $$\mathbf{K}={\upmu (\nabla \mathbf{u}+(\nabla (\mathbf{u}))}^{\mathrm{T}}).$$

5 $$\rho \nabla \cdot \left(\mathbf{u}\right)=0, (\mathrm{Incompressible flow})$$

6 $$\rho {\mathrm{C}}_{\mathrm{p}}\frac{\partial \mathrm{T}}{\partial t}+\rho {C}_{p}\mathbf{u}\cdot \nabla \mathrm{T}+\nabla \cdot {\varvec{q}}={Q}_{p}+{Q}_{vd}+Q.$$

The underlying relationships comprise the Eq. (6)

7 $${\varvec{q}}=-k\nabla T, Q=0, { Q}_{vd}=\tau \cdot \nabla u, { Q}_{p}={\alpha }_{p}T\left(\frac{\partial p}{\partial t}+u\nabla p\right), {\alpha }_{p}=-\frac{1}{p}\frac{\partial p}{\partial t},$$

where $$\tau =-pI+\mu {A}_{1}$$ and $$trace\left(\tau \cdot \nabla \mathrm{u}\right)=\tau .$$

The equations of the functional magnetic field are given as:

8 $$\nabla \times H=J, B=\nabla \times A, J=\sigma E+{J}_{e}, E=-\frac{\partial A}{\partial t}$$

$\nabla \times H=J$ is Ampère's law for magneto-statics. It states that the curl of the magnetic field intensity (H) is equal to the current density (J). $B=\nabla \times A$ relates the magnetic field (B) to the curl of the vector potential (A). $J=\sigma E+{J}_{e}$ describes the current density (J) in terms of two components: conduction current density $\sigma E$ and any additional current density ${J}_{e}$. The first term $\sigma E$ represents the conduction of electric current due to the electric field (E) and the electrical conductivity (σ) of the material. The second term ${J}_{e}$ accounts for any other sources of current density that might be present. $E=-\frac{\partial A}{\partial t}$ represents Faraday's law of electromagnetic induction. It states that the electric field E is equal to the negative rate of change of the vector potential A concerning time t. This law indicates that a changing magnetic field induces an electric field, which is a foundational principle in electromagnetic theory. The thermo-physical properties of blood are given in Table 1.

## Boundary conditions

Following are the specified boundary conditions of the given model^25^.

### Boundary conditions at inlet

At the inlet of the artery, blood velocity is zero. The speed at the intake and the inflow could regulate the arterial pressure.

The boundary condition equation at the inlet was given by the equation:

9 $${u}_{r }(\mathrm{r},\mathrm{ z},\mathrm{ t}) = -{U}_{0}\mathbf{n}$$

### Boundary conditions at outlet

To give the simulations a much more authentic look, we provided the blood supply model's pressure at the outflow. The outlet is on the opposite side of the inlet boundary. The outlet equation is given below:

10 $$\left[-\mathrm{P}\mathbf{I}+\mathbf{K}\right]\mathsf{n}=-\widehat{{\mathrm{P}}_{0}}\mathsf{n},$$

$$\widehat{{\mathrm{P}}_{0}} \le {\mathrm{P}}_{0}.$$

### Boundary conditions at wall

Because of the viscous nature of the blood, it cannot pass through the walls and adheres to it instead. No slip condition is considered here. So the equation at the wall was as follows:

11 $${u}_{r }=0, {u}_{z}=0.$$

### Equation of thermal insulation

All the boundaries of the geometry are thermally insulated. The equation of thermal insulation is given below:

12 $$-\mathbf{n}\cdot \mathbf{q}= 0.$$

### Equation of heat flux

A general inward heat flux of 1200 Wm^2^ is supplied to the inlet of the cylinder. The main equation of heat flux is specified as:

13 $$-\mathbf{n}\cdot \mathbf{q}= {\mathbf{q}}_{0.}$$

### Equation of magnetic insulation:

The equation of magnetic insulation is given by

14 $$\mathsf{n}\times \mathsf{A}=0.$$

## Mathematical formulation

The governing equations for mass, momentum, and energy for the specified velocity field,

$V=\left({{v}_{r}}{^{\prime}}\left(r,\theta ,z,t\right),{{v}_{\theta }}{^{\prime}}\left(r,\theta ,z,t\right),{{v}_{z}}{^{\prime}}\left(\mathrm{r},\uptheta ,\mathrm{z},\mathrm{t}\right)\right)$ is as follows:

### Continuity equation

15 $$\frac{\partial {{v}_{r}}{^{\prime}}}{\partial r}+\frac{1}{r}{{v}_{r}}{^{\prime}}+\frac{1}{r}\frac{\partial {{v}_{\theta }}{^{\prime}}}{\partial \theta }+\frac{\partial {{v}_{z}}{^{\prime}}}{\partial z}=0.$$

### Momentum equations

16 $${\rho }_{hnf}\left( \frac{\partial {{v}_{r}}{^{\prime}}}{\partial \mathrm{t}}+ {{v}_{r}}{^{\prime}} \frac{\partial {{v}_{r}}{^{\prime}}}{\partial \mathrm{r}}+\frac{{{v}_{\theta }}{^{\prime}}}{\mathrm{r}} \frac{\partial {{v}_{r}}{^{\prime}}}{\partial\uptheta }-\frac{{{{v}_{\theta }}{^{\prime}}}^{2}}{\mathrm{r}}+ {{v}_{z}}{^{\prime}} \frac{\partial {{v}_{r}}{^{\prime}}}{\partial \mathrm{z}}\right)=-\frac{\partial p}{\partial r}+\frac{1}{r}\frac{\partial \left(r{\mathcal{T}}_{rr}\right)}{\partial r}+\frac{1}{r}\frac{\partial \left({\mathcal{T}}_{r\theta }\right)}{\partial \theta }-\frac{{\mathcal{T}}_{\theta \theta }}{r}+\frac{\partial \left({\mathcal{T}}_{rz}\right)}{\partial z},$$

17 $${\rho }_{hnf}\left( \frac{\partial {{v}_{\theta }}{^{\prime}}}{\partial \mathrm{t}}+ {{v}_{r}}{^{\prime}} \frac{\partial {{v}_{\theta }}{^{\prime}}}{\partial \mathrm{r}}+\frac{{{v}_{\theta }}{^{\prime}}}{\mathrm{r}} \frac{\partial {{v}_{\theta }}{^{\prime}}}{\partial\uptheta }-\frac{{{v}_{r}}{^{\prime}}{{v}_{\theta }}{^{\prime}}}{\mathrm{r}}+ {{v}_{z}}{^{\prime}} \frac{\partial {{v}_{\theta }}{^{\prime}}}{\partial \mathrm{z}}\right)=-\frac{1}{\mathrm{r}}\frac{\partial \mathrm{p}}{\partial\uptheta }+\frac{1}{{\mathrm{r}}^{2}}\frac{\partial \left({\mathrm{r}}^{2}{\mathcal{T}}_{\mathrm{\theta r}}\right)}{\partial \mathrm{r}}+\frac{1}{\mathrm{r}}\frac{\partial {\mathcal{T}}_{\mathrm{\theta \theta }}}{\partial\uptheta }+\frac{\partial {\mathcal{T}}_{\mathrm{\theta z}}}{\partial \mathrm{z}},$$

18 $${\rho }_{hnf}\left( \frac{\partial {{v}_{z}}{^{\prime}}}{\partial \mathrm{t}}+ {{v}_{r}}{^{\prime}} \frac{\partial {{v}_{z}}{^{\prime}}}{\partial \mathrm{r}}+\frac{{{v}_{\theta }}{^{\prime}}}{\mathrm{r}} \frac{\partial {{v}_{z}}{^{\prime}}}{\partial\uptheta }+ {{v}_{z}}{^{\prime}} \frac{\partial {{v}_{\theta }}{^{\prime}}}{\partial \mathrm{z}}\right)=-\frac{\partial \mathrm{p}}{\partial \mathrm{z}}+\frac{1}{\mathrm{r}}\frac{\partial \left(\mathrm{r}{\mathcal{T}}_{\mathrm{rz}}\right)}{\partial \mathrm{r}}+\frac{1}{\mathrm{r}}\frac{\partial \left({\mathcal{T}}_{\mathrm{\theta z}}\right)}{\partial\uptheta }+\frac{\partial \left({\mathcal{T}}_{\mathrm{zz}}\right)}{\partial \mathrm{z}},$$

where $$\mathcal{T}=\left({\left(\nabla \left(U\right)\right)}^{T}+\nabla \left(U\right)\right)$$.

### Energy equation

19 $${(\rho {C}_{p})}_{hnf}\left(\frac{\partial T}{\partial t}+{{v}_{r}}{^{\prime}}\frac{\partial T}{\partial r}+\frac{{{v}_{\theta }}{^{\prime}}}{r}\frac{\partial T}{\partial \theta }+{{v}_{z}}{^{\prime}}\frac{\partial T}{\partial z}\right)={\mathrm{k}}_{hnf}\left[\frac{1}{r}\frac{\partial }{\partial r}\left(r\frac{\partial T}{\partial r}\right)+\frac{1}{{r}^{2}}\frac{{\partial }^{2}T}{\partial {\theta }^{2}}+\frac{{\partial }^{2}T}{\partial {z}^{2}}\right]+{\mu }_{hnf}\varphi .$$

For a compressible flow, the dissipation function in cylindrical coordinates is denoted by:

$$\varphi =2{\left( \frac{\partial {{v}_{r}}^{{^{\prime}}}}{\partial r}\right)}^{2}+2{\left(\frac{1}{r}\frac{\partial {{v}_{\theta }}^{{{\prime}}}}{\partial \theta }+\frac{{{v}_{r}}^{{{\prime}}}}{r}\right)}^{2}+2{\left( \frac{\partial {{v}_{z}}^{{{\prime}}}}{\partial z}\right)}^{2}+{\left( \frac{\partial {{v}_{\theta }}^{{{\prime}}}}{\partial r}-\frac{{{v}_{\theta }}^{{{\prime}}}}{r}+\frac{1}{r} \frac{\partial {{v}_{r}}^{{^{\prime}}}}{\partial \theta }\right)}^{2}+{\left(\frac{1}{r}\frac{\partial {{v}_{z}}^{{{\prime}}}}{\partial \theta }+\frac{\partial {{v}_{\theta }}^{{{\prime}}}}{\partial z}\right)}^{2}+{\left( \frac{\partial {{v}_{r}}^{{{\prime}}}}{\partial z}+\frac{\partial {{v}_{z}}^{{{\prime}}}}{\partial r}\right)}^{2}.$$

After applying the velocity vector U = [${{v}_{r}}{^{\prime}}(r, z, t),0, {{v}_{z}}{^{\prime}}(r,z, t)]$, and uniform magnetic field the above equation reduces to the following equations

20 $$\frac{\partial {{v}_{r}}{^{\prime}}}{\partial r}+\frac{{{v}_{r}}{^{\prime}}}{r}+\frac{\partial {{v}_{z}}{^{\prime}}}{\partial z}=0,$$

21 $$\left(\frac{\partial {{v}_{r}}{^{\prime}}}{\partial t}+ {{v}_{r}}{^{\prime}}\frac{\partial {{v}_{r}}{^{\prime}}}{\partial r}+ {{v}_{z}}{^{\prime}}\frac{\partial {{v}_{r}}{^{\prime}}}{\partial z}\right)=-\frac{1}{\rho }\frac{\partial p}{\partial r}+{\nu }_{hnf}\left(\frac{{\partial }^{2}{{v}_{r}}{^{\prime}}}{\partial {r}^{2}}+\frac{1}{r}\right. \frac{\partial {{v}_{r}}{^{\prime}}}{\partial r}+\frac{{\partial }^{2}{{v}_{r}}{^{\prime}}}{\partial {z}^{2}}\left.-\frac{{{v}_{r}}{^{\prime}}}{{r}^{2}}\right)+g({\rho \gamma )}_{hnf}\alpha \left(T-{T}_{0}\right)-\sigma {{B}_{0}}^{2}{{v}_{r}}{^{\prime}},$$

22 $$\frac{\partial p}{\partial \theta }=0,$$

23 $$\left(\frac{\partial {{v}_{z}}{^{\prime}}}{\partial t}+ {{v}_{r}}{^{\prime}}\frac{\partial {{v}_{z}}{^{\prime}}}{\partial r}+ {{v}_{z}}{^{\prime}}\frac{\partial {{v}_{z}}{^{\prime}}}{\partial z}\right)=-\frac{1}{\rho }\frac{\partial p}{\partial z}+{\nu }_{hnf}\left(\frac{{\partial }^{2}{{v}_{z}}{^{\prime}}}{\partial {r}^{2}}+\frac{{\partial }^{2}{{v}_{z}}{^{\prime}}}{\partial {z}^{2}}+\frac{1}{r}\frac{\partial {{v}_{z}}{^{\prime}}}{\partial r}\right)+g({\rho \gamma )}_{hnf}\alpha \left(T-{T}_{0}\right)-\sigma {{B}_{0}}^{2}{{v}_{z}}{^{\prime}},$$

24 $${(\rho {C}_{p})}_{hnf}\left(\frac{\partial T}{\partial t}+{{v}_{r}}{^{\prime}}\frac{\partial T}{\partial r}+{{v}_{z}}{^{\prime}}\frac{\partial T}{\partial z}\right)={K}_{hnf}\left(\frac{1}{r}\frac{\partial T}{\partial r}+\frac{{\partial }^{2}T}{\partial {r}^{2}}+\frac{{\partial }^{2}T}{\partial {z}^{2}}\right)+{Q}_{0}.$$

where ${\rho }_{hnf}$ is the density, ${\nu }_{hnf}$ is the kinematic viscosity of the hybrid nano-fluid and T denotes the absolute temperature. The specific heat capacity and thermal conductivity of nanoparticles are ${\left(\rho {C}_{p}\right)}_{hnf}$ and ${K}_{hnf}$, respectively.

The following are the thermo-physical characteristics of hybrid nanoparticles^26^:

25 $$\left.\begin{array}{l}{\rho }_{hnf}= \left(1-{\phi }_{2}\right)\left( \left(1-{\phi }_{1}\right)\right.{\rho }_{f}+{\phi }_{1} {\rho }_{{s}_{1}})+{\phi }_{2}{\rho }_{{s}_{2}},\\ {(\rho {C}_{p})}_{hnf}=\left(1-{\phi }_{2}\right){(\left(1-{\phi }_{1}\right)\rho {C}_{p})}_{f}+{\phi }_{1}{(\rho {C}_{p})}_{{s}_{1}})+{\phi }_{2}{(\rho {C}_{p})}_{{s}_{2}},\\ {\mu }_{hnf}= \frac{{\mu }_{f}}{{\left(1-{\phi }_{1}\right)}^{2.5}{\left(1-{\phi }_{2}\right)}^{2.5}},\\ \frac{{K}_{hnf}}{{K}_{f}}= \left\{\frac{{k}_{{s}_{1}+}2{k}_{f}-2{\phi }_{1}\left({k}_{f}-{k}_{{s}_{1}}\right)}{{k}_{{s}_{1}+}2{k}_{f}+{\phi }_{1}\left({k}_{f}-{k}_{{s}_{1}}\right)}\times \frac{{k}_{{s}_{2}+}2{k}_{nf}-2{\phi }_{2}\left({k}_{nf}-{k}_{{s}_{2}}\right)}{{k}_{{s}_{2}+}2{k}_{nf}+{\phi }_{2}\left({k}_{nf}-{k}_{{s}_{2}}\right)}\right\}.\end{array}\right\}.$$

## Mesh description

Among the key components of computational fluid dynamics is mesh. The mesh's efficiency can be used to gauge the number of iterations and the solution's precision.

Finite element mesh serves two purposes:

- Represents the geometry
- Represents the solution field

**Figure 2:**
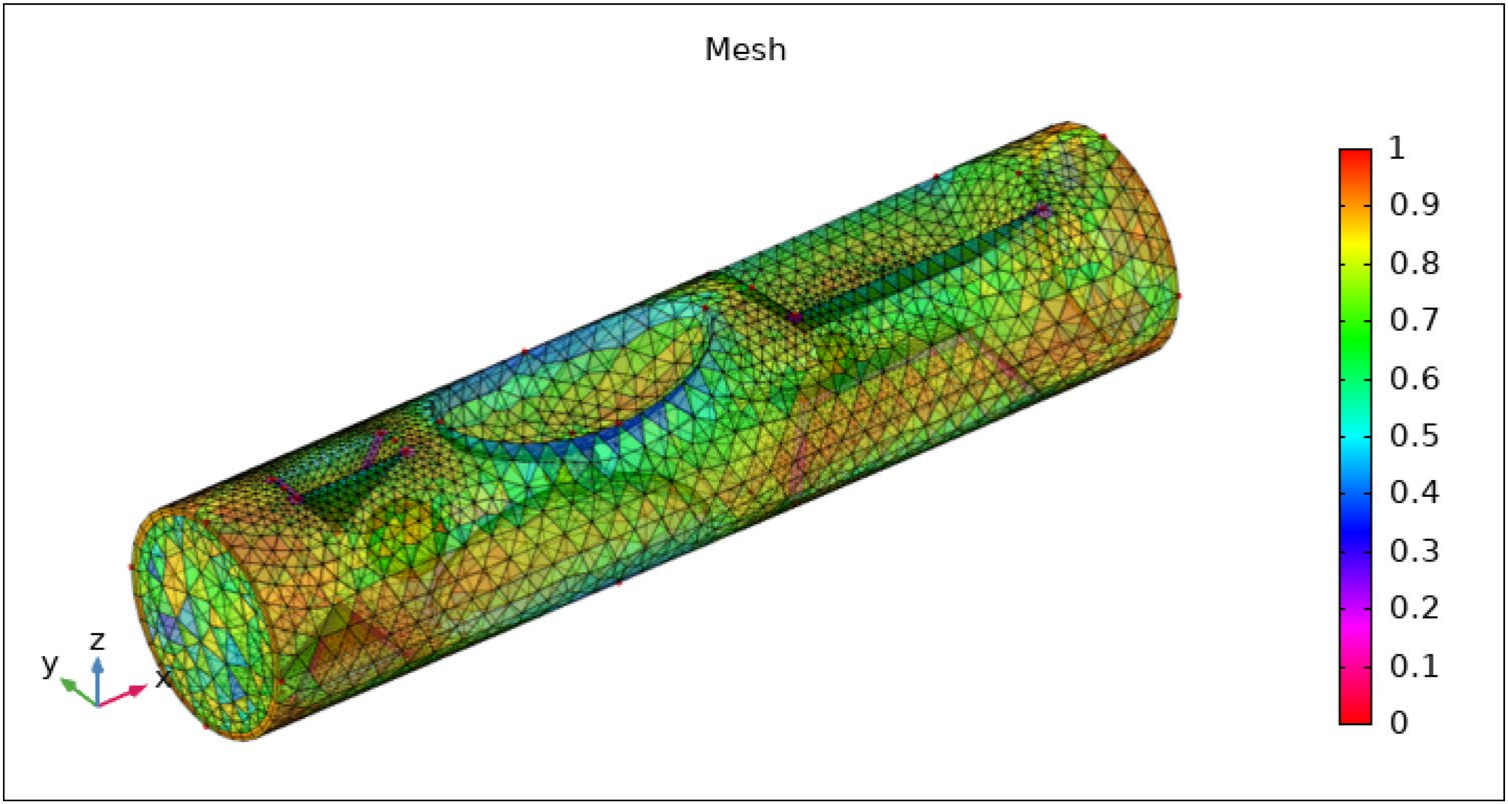
Finite element mesh of geometry.

The finite element discretization of the geometry follows the same principles as the discretization of the system's mathematical model, the result is approximated as continuous across each couple of points in the mesh. A mesh with finer components creates more precise outcomes and offers superior functionality. It is noted that the mesh is extra rectified in the stenotic section, as can be observed by the stenotic area, and less rectified while far distant from the stenosis, as seen in Fig. 2. The elucidation of the size of the mesh is also given in Table 2. The other information about the mesh stats is given below in Tables 3 and 4.

**Table 1:** Numerical data of blood, gold, and silver nanoparticles^27^.

Property	Heat capacity $\left({\mathrm{JK}}^{-1} {\mathrm{kg}}^{-1}\right)$	Thermal conductivity $\left(\mathrm{W }{\mathrm{m}}^{-1} {\mathrm{k}}^{-1}\right)$	Dynamic viscosity $\left(\mathrm{N }{\mathrm{m}}^{-2}\mathrm{ s}\right)$	Density $\left(\mathrm{kg }{\mathrm{m}}^{-3}\right)$
Blood	3746	0.52	0.003	1063
Gold (Au)	129	310	0.00464	19,300
Silver (Ag)	235	429	0.005	10,500

**Table 2:** Description of mesh dimensions.

Geometric constituents units	Bounds	Geometric constituents units	Bounds
Illustrate for	Fluid dynamics	Minimum element size	0.0522
Resolution of narrow region	0.5	Determination of confined zone	0.7
Maximum element growth rate	1.5	Curvature factor	0.6
Maximum element size	0.29	Predefined size	Normal

**Table 3:** Description of mesh dimensions.

Characteristics	Values
Number of elements	105,407
Total tetrahedrons	90,003
Total prisms	14,426
Total triangles	8418
Total quads	116
Pyramids	978
Vertex elements	66
Edge elements	830
Average element quality	0.6586
Minimum element quality	0.01011
Mesh vertices	24,607
Element volume ratio	3.115E−5
Mesh volume	${0.6426 \mathrm{m}}^{3}$

**Table 4:** Variation in velocity, pressure, and temperature in XZ plane at Y = 0.

Time (t)	Maximum velocity (m ${\mathrm{s}}^{-1}$)	Minimum velocity (m ${\mathrm{s}}^{-1}$)	Maximum pressure (pa)	Minimum pressure (pa)	Maximum temperature (K)	Maximum temperature (K)
0 s	0.57	0	5.57 $\times {10}^{7}$	6.76 $\times {10}^{5}$	313	314
1 s	0.59	0	1.26 $\times {10}^{4}$	5.06 $\times {10}^{3}$	311	314
2 s	0.56	0	1.26 $\times {10}^{4}$	5.16 $\times {10}^{3}$	310	315
3 s	0.56	0	1.27 $\times {10}^{4}$	5.1 $\times {10}^{3}$	308	315
4 s	0.56	0	1.28 $\times {10}^{4}$	5.17 $\times {10}^{3}$	307	316
5 s	0.55	0	1.29 $\times {10}^{4}$	5.33 $\times {10}^{3}$	306	316
6 s	0.55	0	1.3 $\times {10}^{4}$	5.44 $\times {10}^{3}$	304	316
7 s	0.55	0	1.31 $\times {10}^{4}$	5.46 $\times {10}^{3}$	304	317
8 s	0.55	0	1.31 $\times {10}^{4}$	5.48 $\times {10}^{3}$	303	317
9 s	0.55	0	1.31 $\times {10}^{4}$	5.48 $\times {10}^{3}$	302	317
10 s	0.55	0	1.31 $\times {10}^{4}$	5.49 $\times {10}^{3}$	301	317

## Graphical results and discussion

The influence of induced magnetic field and hybrid nanoparticles (silver and gold) on blood flow in defective arteries with multiple stenoses on the outer layers and erratic thrombus (clots) of different radii via the inner part of the artery is deliberated in this study using 3-D computational simulation. The experiment is performed on a blood conduit with mild stenosis assumptions, and expressions of temperature, resistance impedance to flow, velocity, wall shear stress, and pressure gradient are generated by employing related boundary conditions. According to the outcomes, the form of stenosis and thrombus at the center of an artery is the primary source of higher shear rates at the arterial walls. The findings support the cardiovascular properties of arteries with stenosis and thrombosis. The addition of hybridized nanoparticles modified the physical characteristics of blood, such as density, ability to conduct heat, electrical conductivity, and dynamic viscosity, influencing the simulation findings. We took a cut plane down the length of the artery in the XZ direction at Y = 0 and discussed it at various intervals.

### Surface velocity, pressure, and temperature assessment

**Figure 3:**
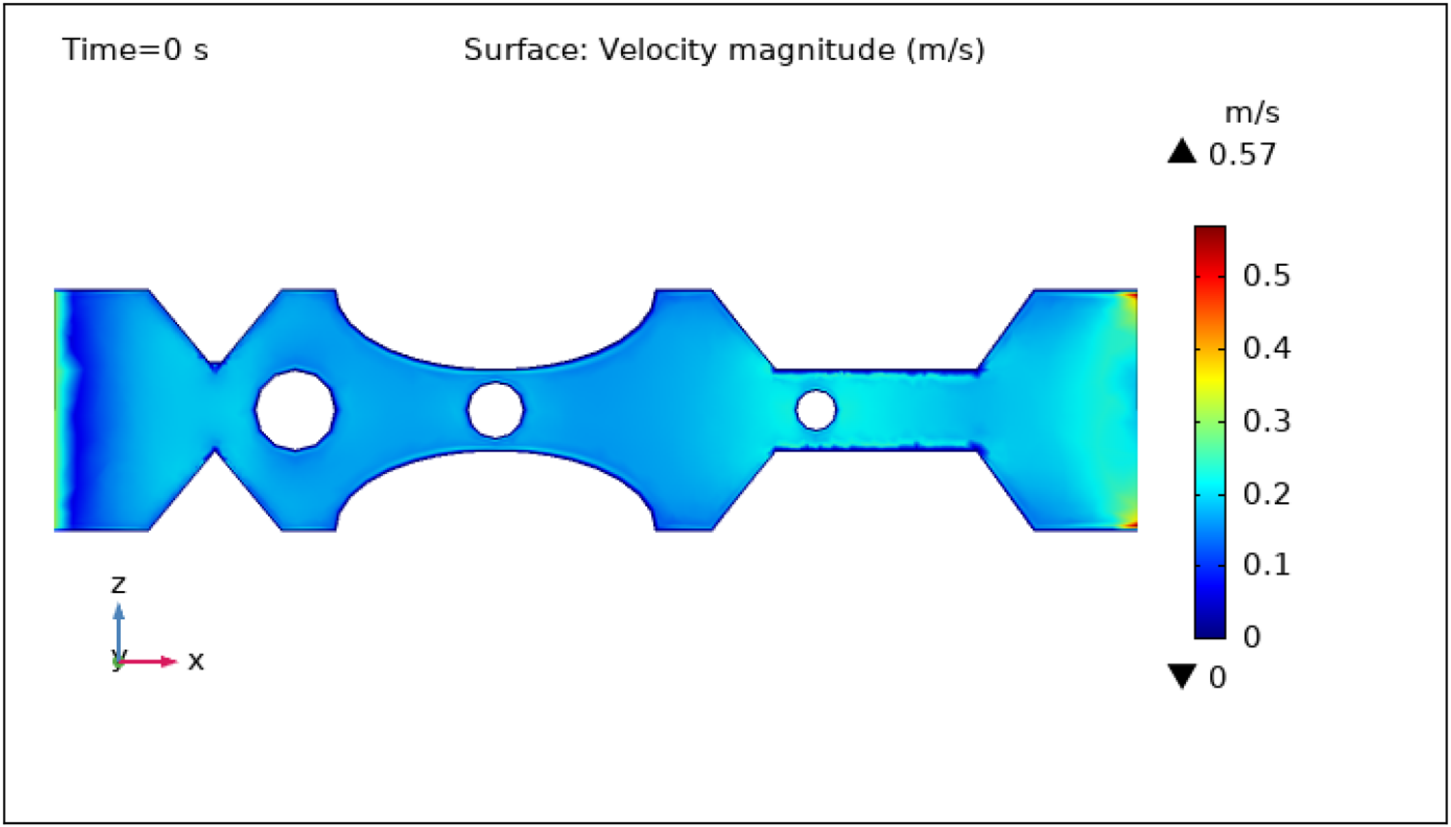
Velocity profile in XZ cut plane at $Y=0$ for $t=0\,{\mathrm{s}}$.

**Figure 4:**
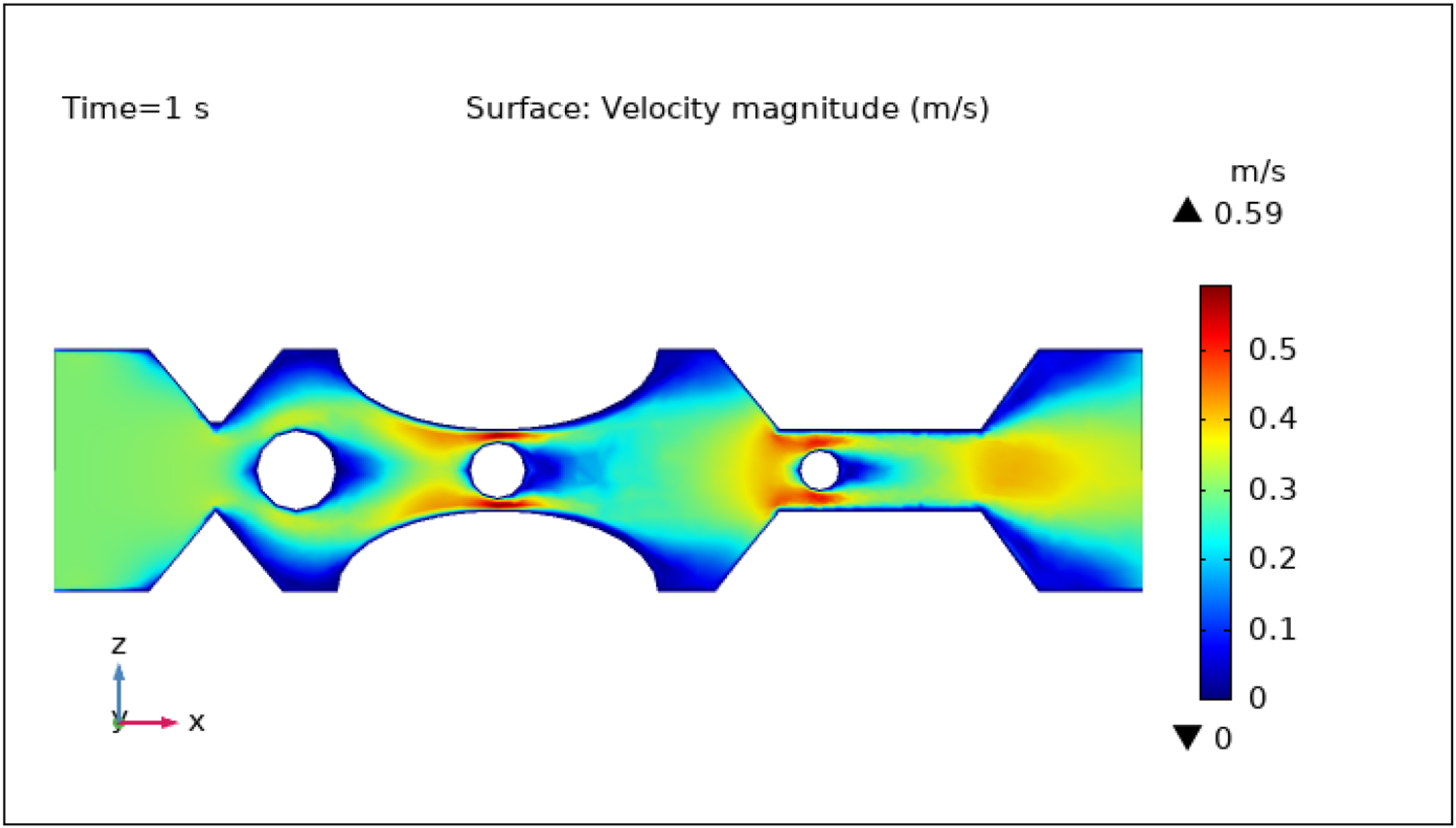
Velocity profile in XZ cut plane at $Y=0$ for $t=1\,\text{s}$.

**Figure 5:**
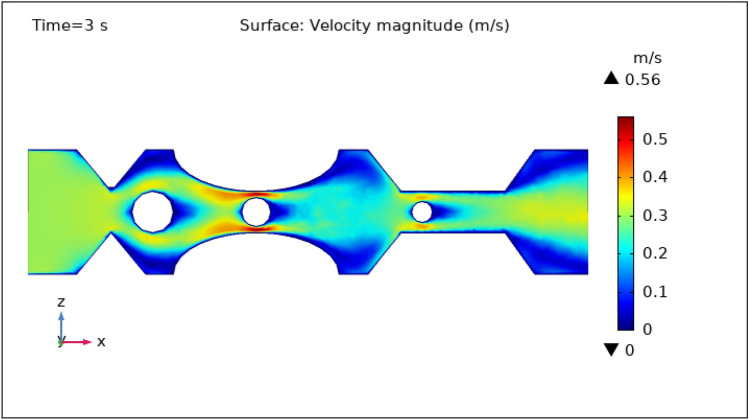
Velocity profile in XZ cut plane at $Y=0$ for $t=3\,\text{s}$.

**Figure 6:**
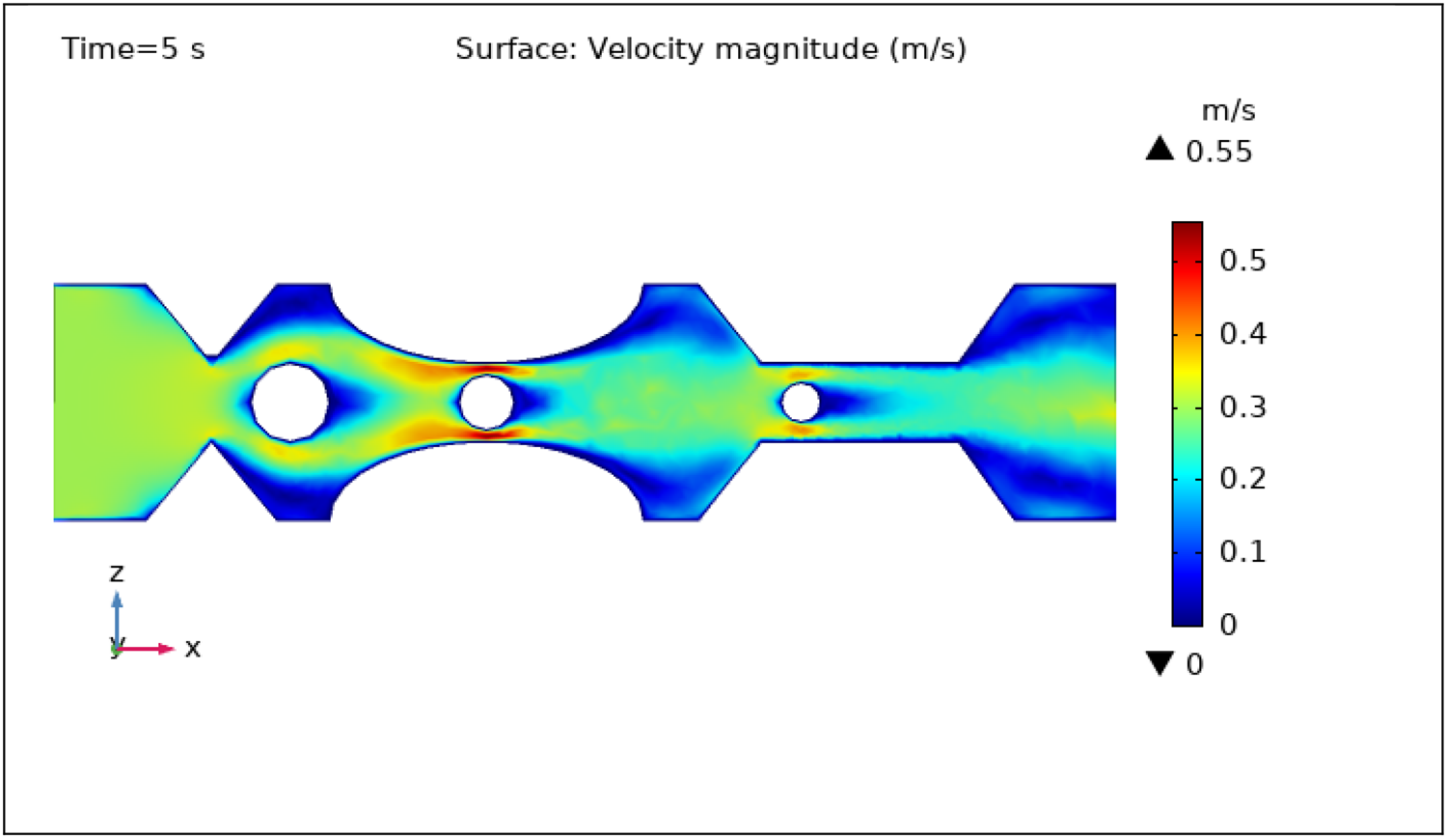
Velocity profile in XZ cut plane at $Y=0$ for $t=5\,\text{s}$.

**Figure 7:**
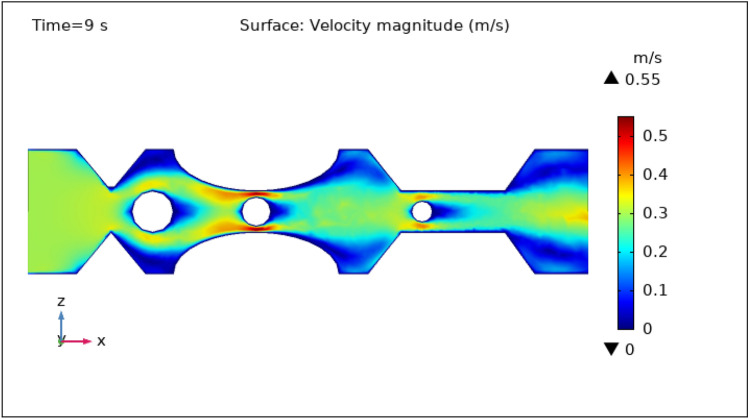
Velocity profile in XZ cut plane at $Y=0$ for $t=9\,\text{s}$.

Figures 3, 4, 5, 6 and 7 show the change in the magnitude of the velocity distribution at different time intervals of 0 s, 1 s, 3 s, 5 s, and 9 s. Figure 3 displays the surface and contour velocity across three impedance shapes at the artery's walls and the erratic thrombus of various radii at the center for 0 s in the presence of magnetized hybrid nanoparticles. At this moment, the greatest velocity is 0.57 m s^−1^ and the minimum velocity is 0 m s^−1^. For 0 s there is no change in velocity along the artery's length. Figure 4 shows the maximum velocity is $0.59 \mathrm{m }{\mathrm{s}}^{-1}$ at 1 s. At this moment, the greatest velocity is 0.59 $\mathrm{m }{\mathrm{s}}^{-1}$ along the artery's length in the area of stenosis where there is are thrombus, and the minimum velocity is at the boundary walls, as indicated by the legend. The location where the velocity dramatically increases to 0.59 $\mathrm{m}{\mathrm{ s}}^{-1}$ is due to thrombus and wall obstruction, which causes blood to have less space to flow and its speed to increase, as a result, pressure to elevate at the boundary, which can cause the artery to burst. Hybrid nanoparticles can interact with plaque or blockages in the arterial lumen when injected into stenosed arteries. Nanoparticles may aggregate on the plaque surface or enter into the constricted zone, depending on their size and properties. The blood flow improves due to the addition of magnetized hybrid nanoparticles as compared to when we did not deliver nanoparticles in the artery. The magnetic nanoparticles manage the pressure at the boundary walls, lowering the possibility of the artery wall collapsing. It is worth noting that when the free-flowing flow departs the stenotic artery, the flow pattern scatters, and the broadest surface magnitude is observed at the top and lower limits of the stenotic artery. It is critical to understand the influence of hybrid nanoparticles on blood velocity in stenosed and thrombosed arteries to design targeted therapeutics. These nanoparticles are engineered to target specific obstructions or clots and deliver therapeutic medicines or apply mechanical forces to lessen or dissolve the clots. Figure 5, 6 and 7 delineates the velocity at 3 s, 5 s, and 9 s. The maximum velocity for 3 s is 0.56 $\mathrm{m }{\mathrm{s}}^{-1}$ at the center of the artery. The maximum and minimum values for 5 s and 9 s are 0.55 $\mathrm{m}{\mathrm{ s}}^{-1}$ and 0 $\mathrm{m }{\mathrm{s}}^{-1}$, respectively. This finding supports the idea that as blood flows across the narrow section, the pressure applied on the artery walls rises swiftly. It is also noteworthy that the surface velocity patterns are symmetrical throughout the arterial length.

**Figure 8:**
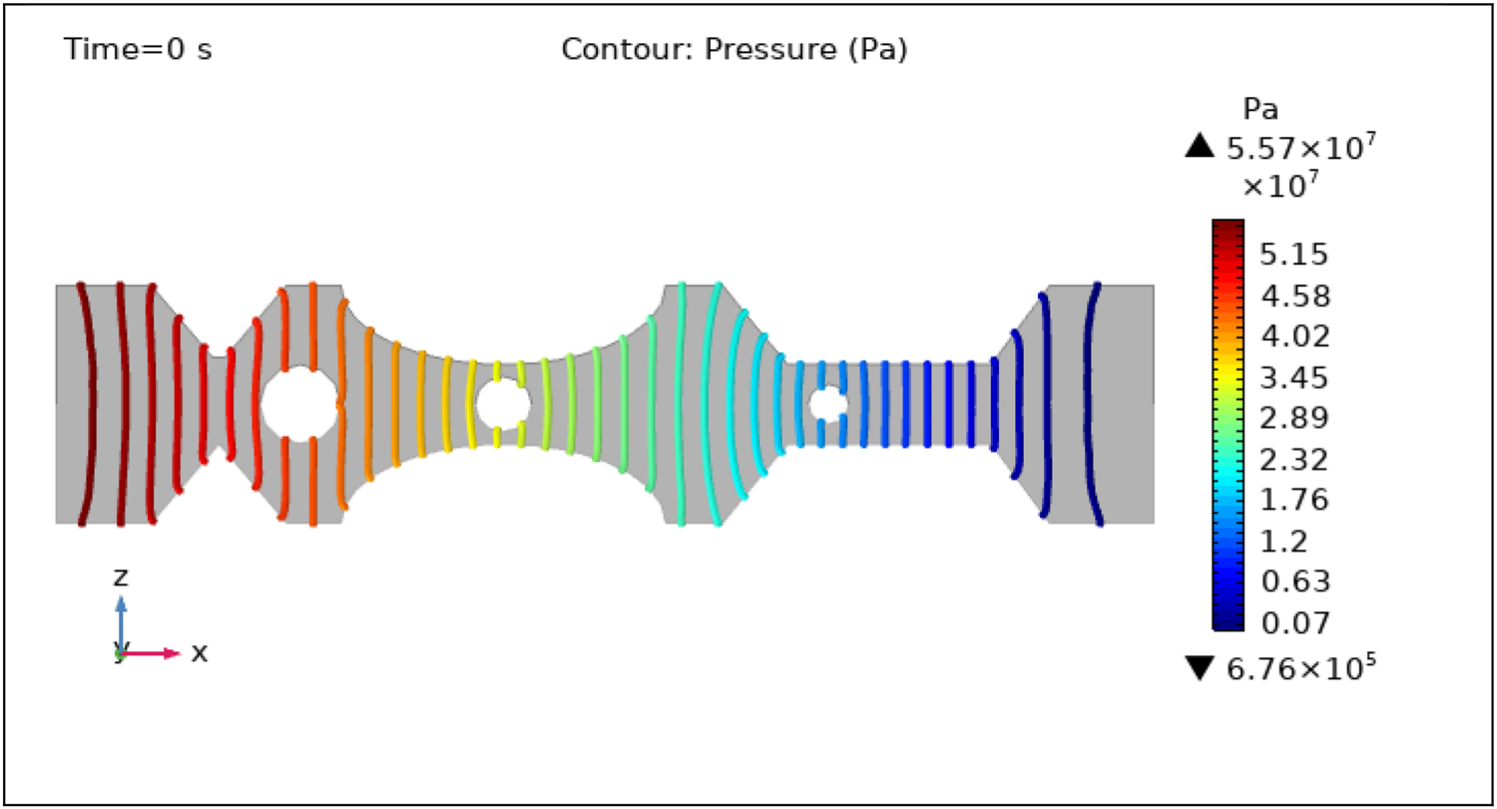
Contour pressure profile in XZ cut plane at $Y=0$ for $t=0\,\text{s}$.

**Figure 9:**
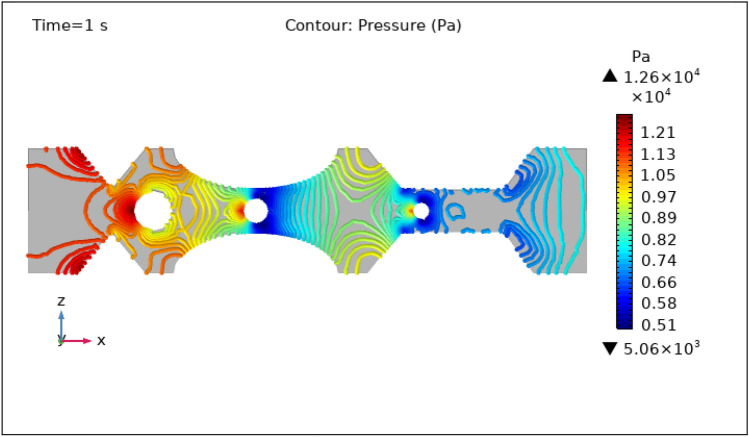
Contour pressure profile in XZ cut plane at $Y=0$ for $t=1\,\text{s}$.

**Figure 10:**
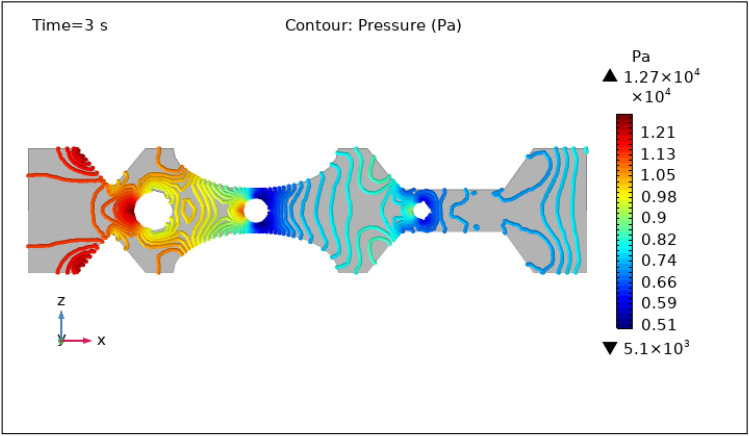
Contour pressure profile in XZ cut plane at $Y=0$ for $t=3\,\text{s}$.

**Figure 11:**
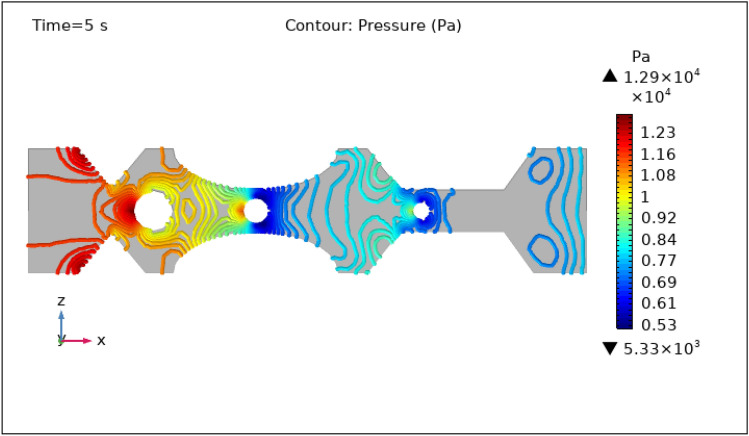
Contour pressure profile in XZ cut plane at $Y=0$ for $t=5\,\text{s}$.

**Figure 12:**
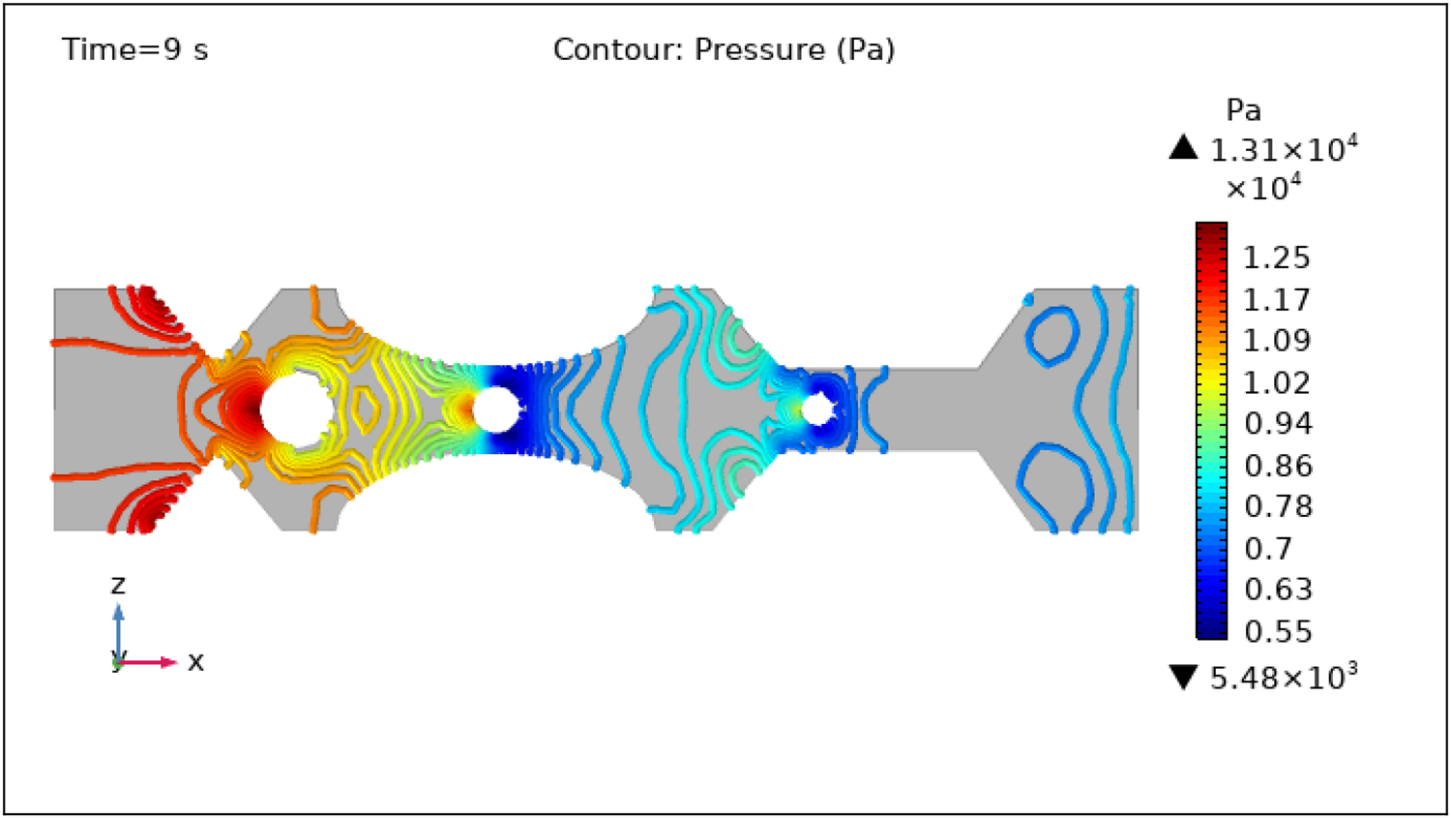
Contour pressure profile in XZ cut plane at $Y=0$ for $t=9\,\text{s}$.

Figures 8, 9, 10, 11 and 12, depict the magnitude of pressure exerted by blood at various periods of 0 s, 1 s, 3 s, 5 s, and 9 s in 3-D. Aside from blood velocity, the presence of hybrid nanoparticles affect wall shear stress and rate in stenosed and thrombosed arteries. The frictional forces exerted by flowing blood on the inner walls of blood arteries are characterized by wall shear stress and wall shear rate. Figure 8 demonstrates the pressure resistance at the walls of a restricted artery due to fluid movement during 0 s. It is worth noting that the pressure gradient reaches a high of $5.57\times {10}^{7}$ pa and a minimum of $6.76\times {10}^{5}$ pa for 0 s. Figure 9 delineates the pressure of hybrid nano-fluid at time 1 s. The maximum value of pressure at this period is $1.26\times {10}^{4}$ pa and the minimum is $5.06\times {10}^{3}$ pa throughout the artery. The existence of hybrid nanoparticles may potentially affect wall shear stress. The interaction of nanoparticles with the thrombus might influence the clot's stability and shape, potentially causing changes in local flow patterns and wall shear stress distribution. The presence of nanoparticles within a thrombus may change the dynamic balance between blood flow and thrombus stability, influencing the stresses exerted on artery walls. The decline in pressure is attributed to the injection of magnetized hybrid nanoparticles, as the pressure was substantially greater before we delivered the nanoparticles. Figures 10, 11 and 12, depict the blood pressure for 3, 5, and 9 s, respectively. Legends show the highest and lowest values for all other times and the type of pressure graphs in all circumstances. The graphs are all symmetrical and pressure readings vary depending on location as well as time. These findings indicate that including hybrid nanoparticles and a magnetic field reduces pressure at the boundary and boosts blood flow.

**Figure 13:**
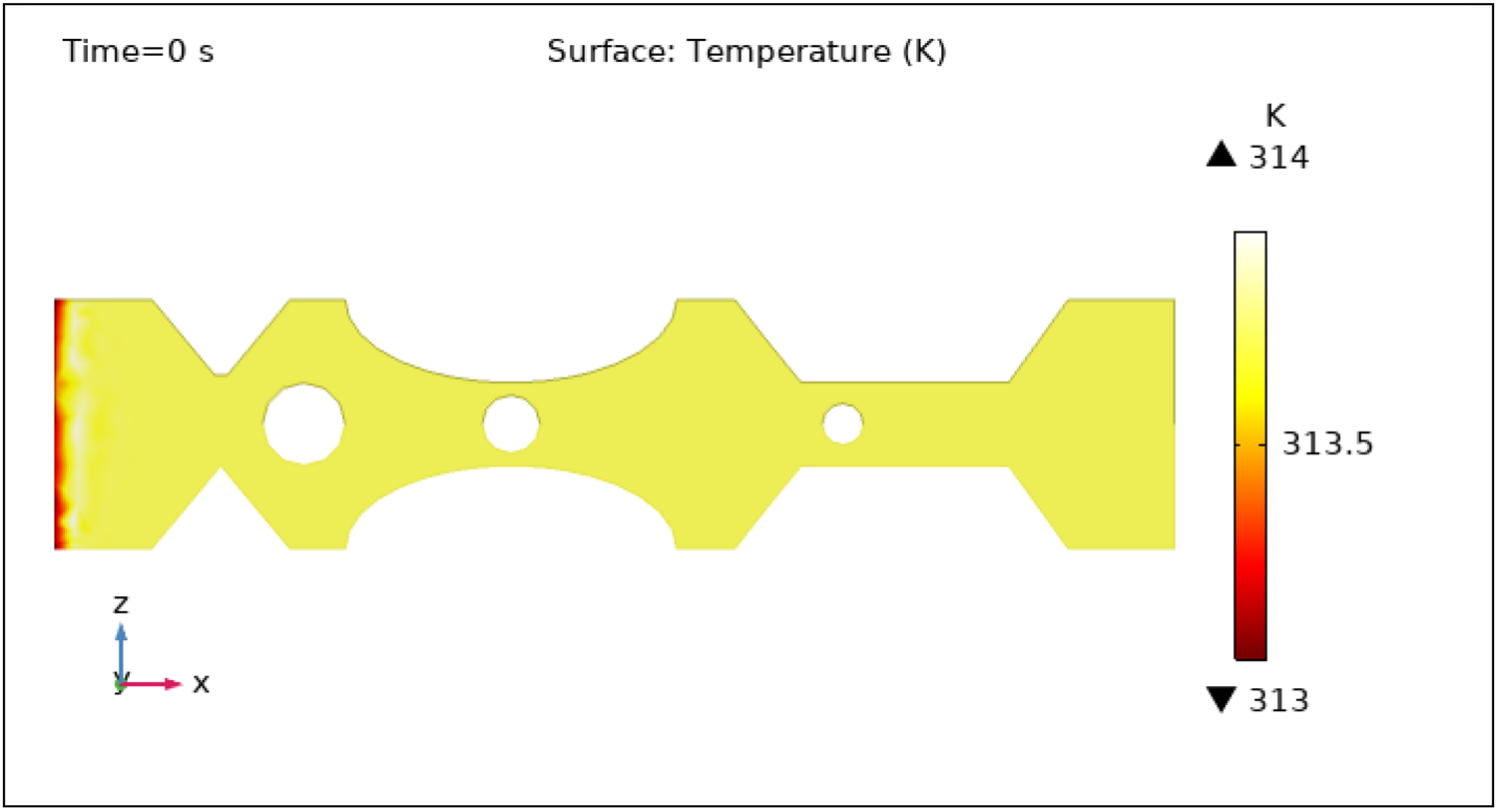
Surface temperature profile in XZ cut plane at $Y=0$ for $t=0\,\text{s}$.

**Figure 14:**
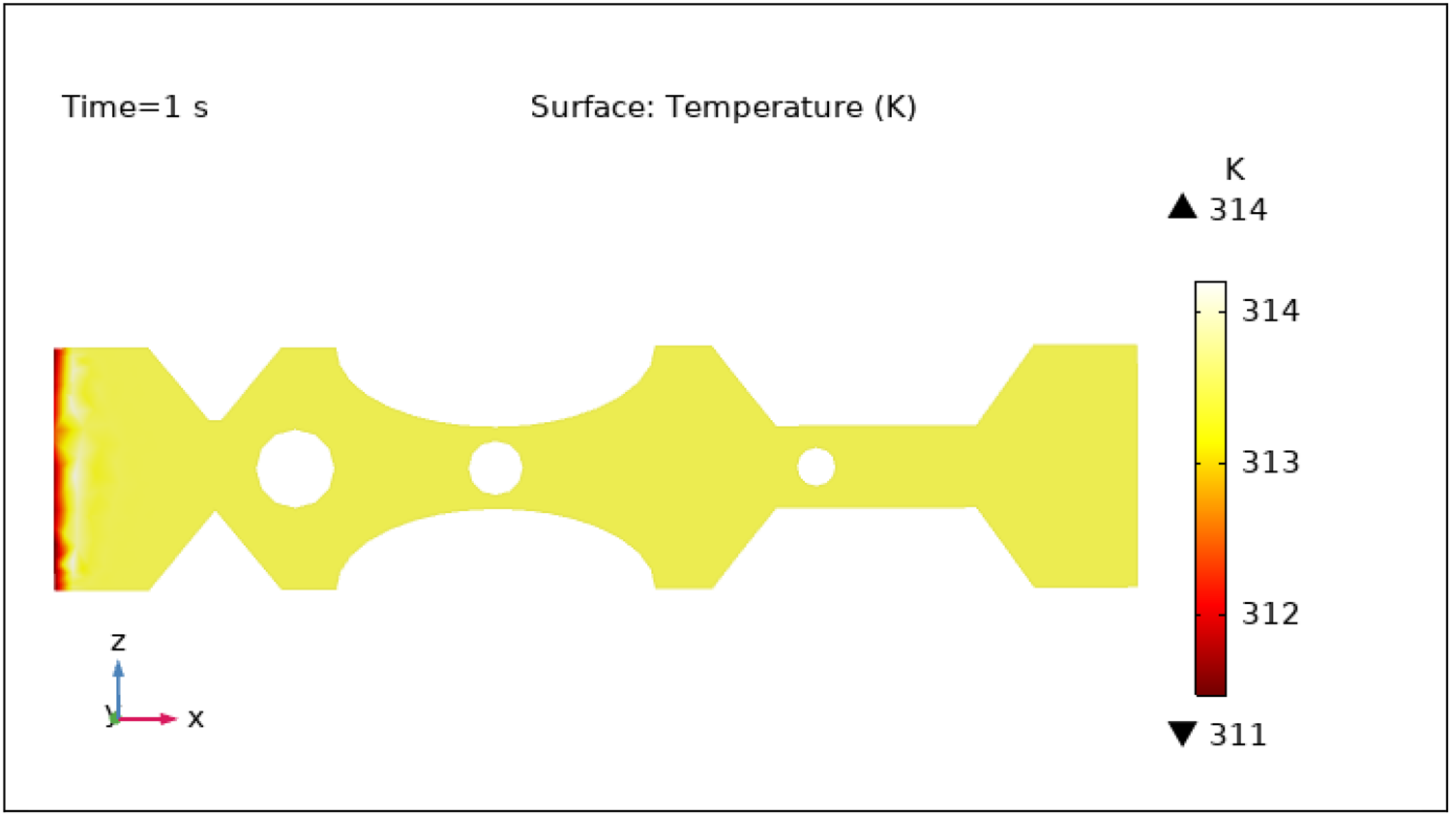
Surface temperature profile in XZ cut plane at $Y=0$ for $t=1\,\text{s}$.

**Figure 15:**
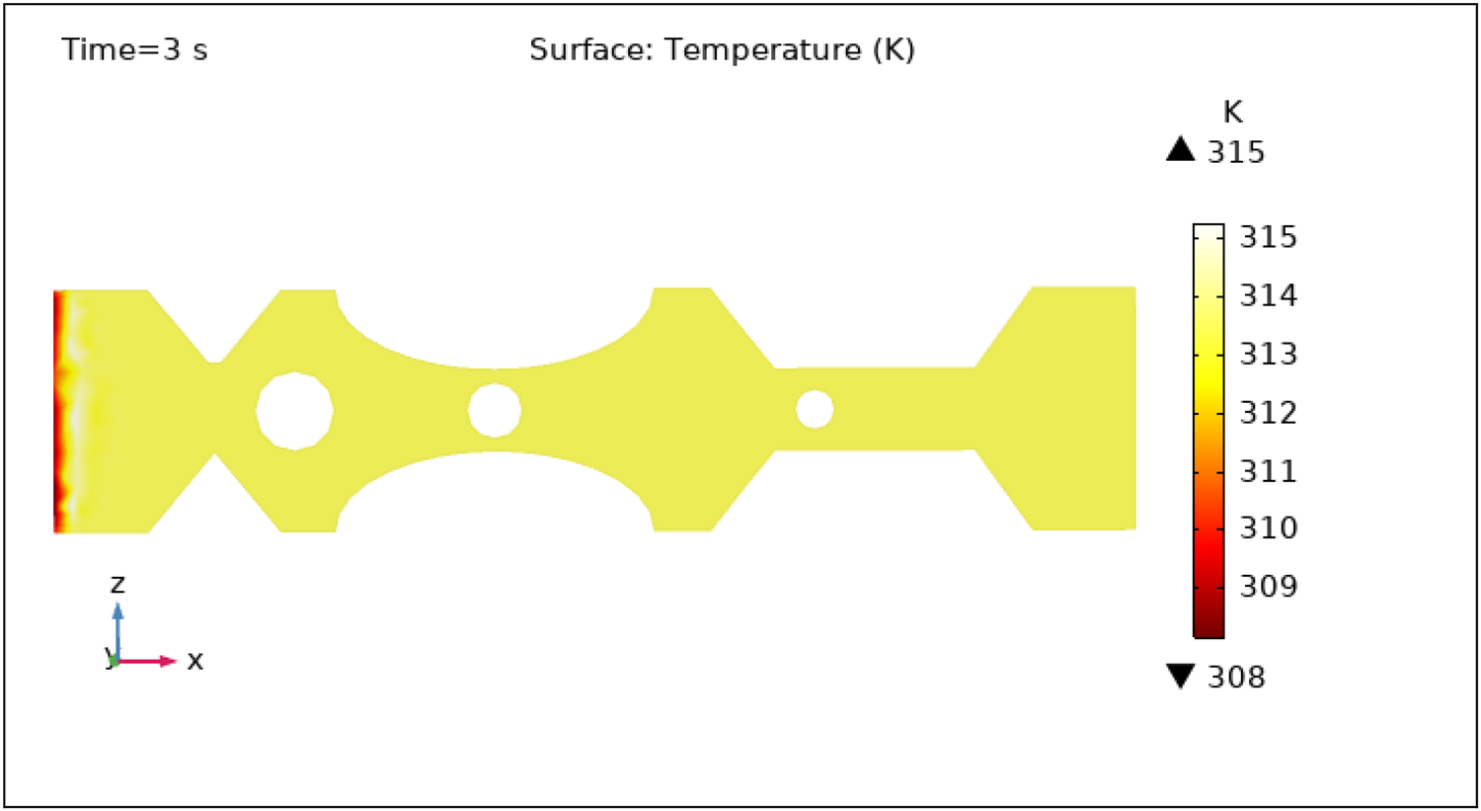
Surface temperature profile in XZ cut plane at $Y=0$ for $t=3\,\text{s}$.

**Figure 16:**
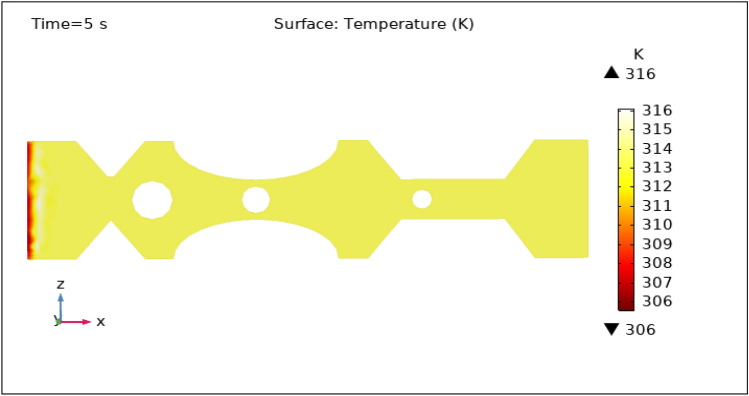
Surface temperature profile in XZ cut plane at $Y=0$ for $t=5\,\text{s}$.

**Figure 17:**
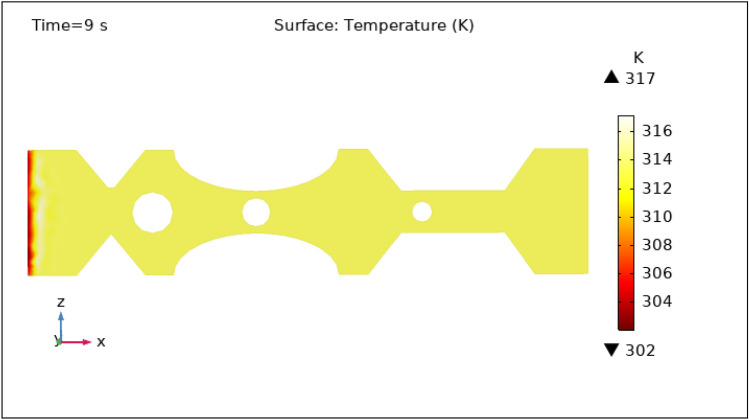
Surface temperature profile in XZ cut plane at $Y=0$ for $t=9\,\text{s}$.

Another important characteristic controlled by hybrid nanoparticles and the induced magnetic field is temperature. When nanoparticles are subjected to an alternating magnetic field, they create heat via mechanisms such as hysteresis losses and magnetic relaxation. The temperature of the surrounding blood and tissue can be raised as a result of this localized heating. To avoid any negative effects on healthy tissues and to achieve the intended therapeutic outcomes, the temperature is carefully monitored and controlled by specific hybrid nanoparticles volume fraction. Figures 13, 14, 15, 16 and 17 show the magnitude of temperature implemented by nano-fluid at 0 s, 1 s, 3 s, 5 s, and 9 s. For 0 s the maximum temperature is 314 K and the minimum temperature is 313 K. With time the minimum temperature is reduced to 311 K and the maximum temperature remains the same. We can examine from the graphs below as time increases the minimum temperature reduced rapidly as compared to the maximum temperature which increased by 3 K only. The temperature is highest at the intake and remains almost constant throughout the stenotic artery. The variation in the temperature is observed only at the inlet of the artery.

## Visualization of line graphs

The line graph depicts the behavior of blood flow and temperature all along the artery.

**Figure 18:**
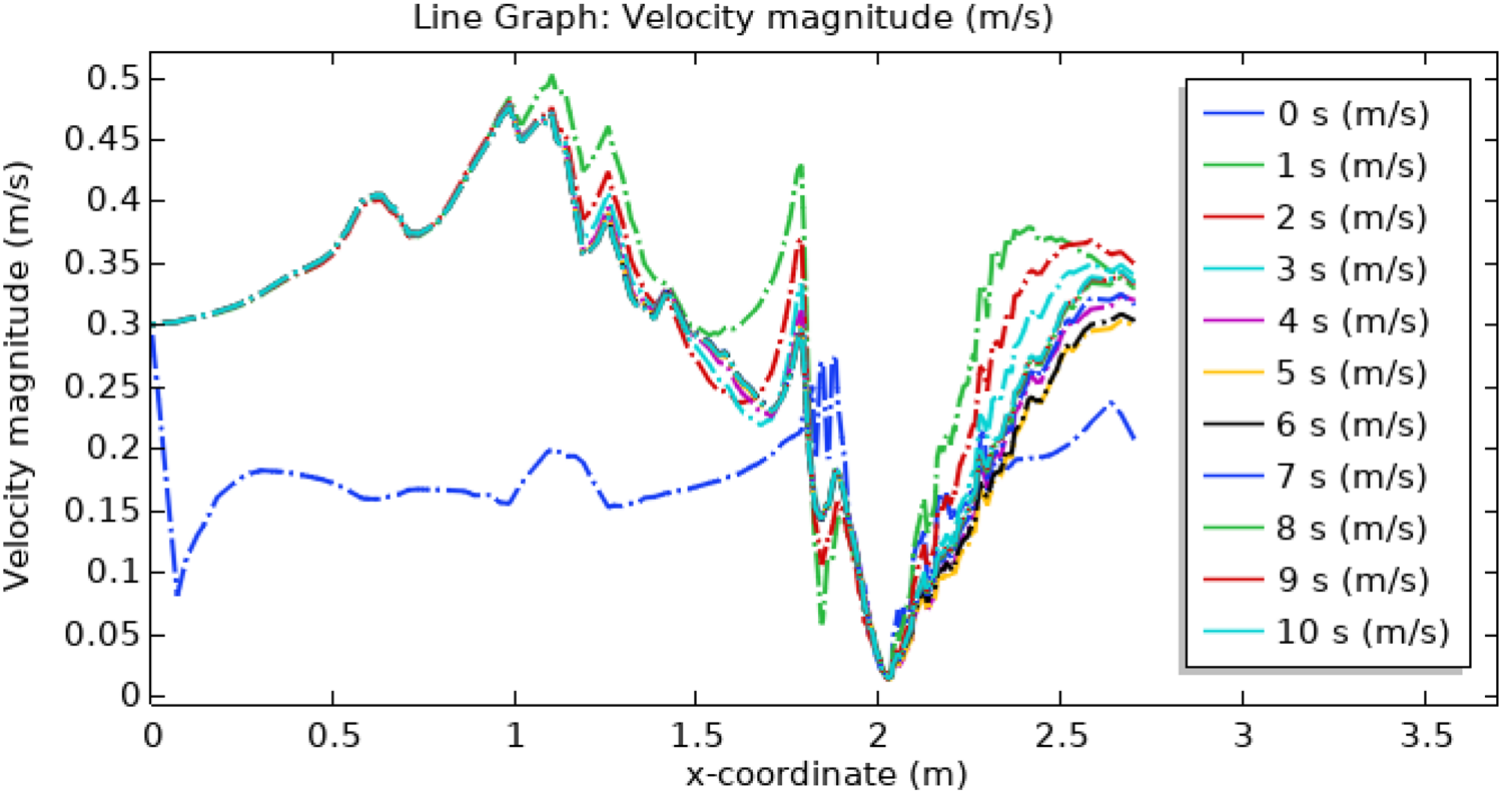
Line graph of blood velocity with magnetized nanoparticles.

**Figure 19:**
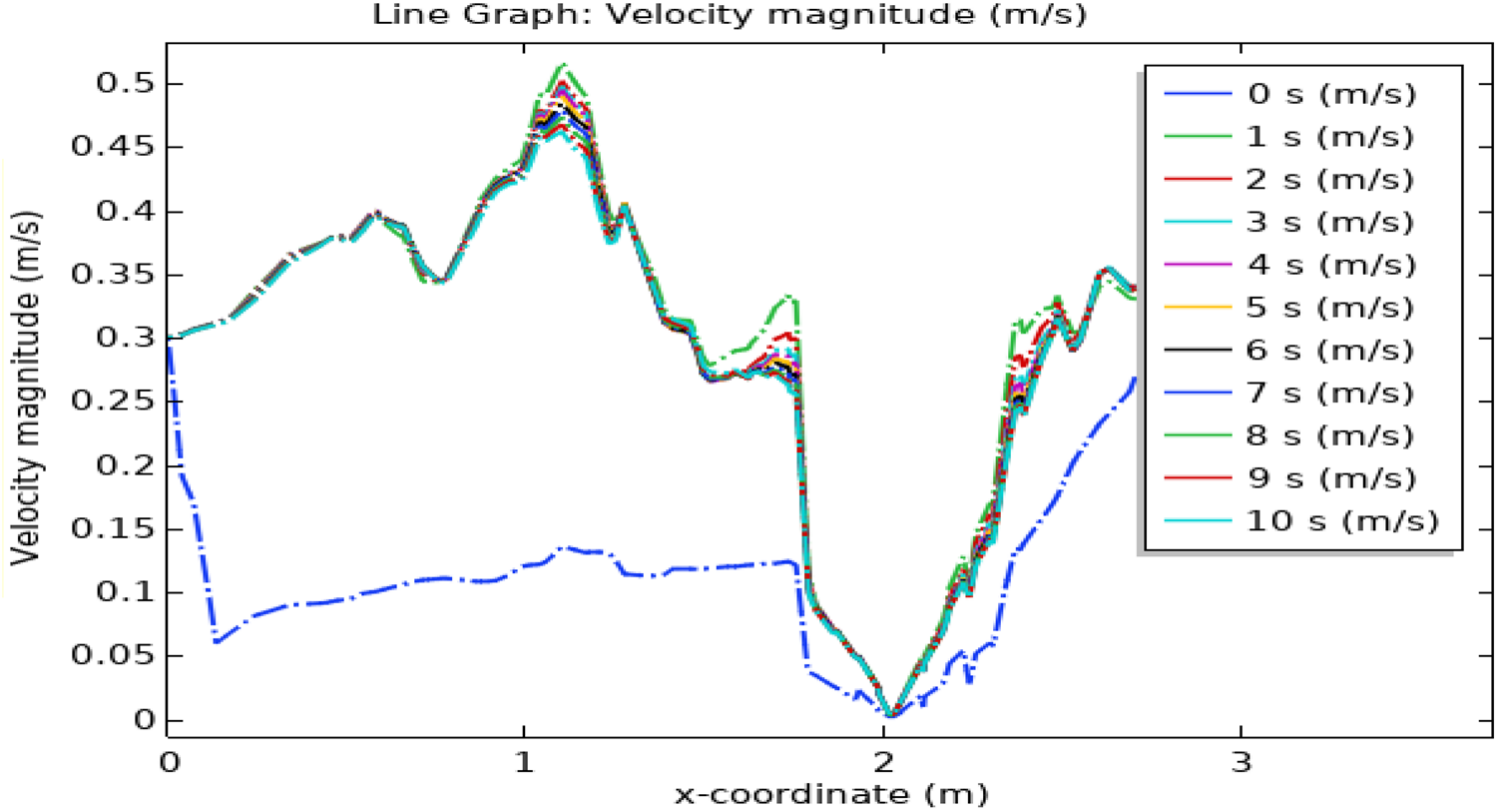
Line graph of blood velocity without magnetized nanoparticles.

Figure 18 illustrates the velocity profile concerning position along the x-axis and time in the artery. At the start, the blood is flowing with normal velocity in the non-stenotic region. Just as the triangular stenosis starts the velocity slightly increases. Just after blood exits triangular stenosis there is a thrombus of 0.1 m radius which blocks the blood flow due to which its velocity abruptly increases till 0.57 m ${\mathrm{s}}^{-1}.$ In the region of elliptical stenosis and thrombus of radius 0.05 m, the velocity reaches its maximum value of 0.45 m ${\mathrm{s}}^{-1}$. In the region of trapezoidal stenosis and thrombus of radius 0.07 m velocity drops to 0.34 m s^−1^. Hence we conclude that as time increases velocity along the x-axis also increases. So line graph of the velocity profile shows that the velocity is maximum for elliptical stenosis and is minimum for trapezoidal-shaped stenosis. The legend shows the change in velocity concerning time. Figure 19 elucidates the blood velocity dynamics in the absence of magnetized hybrid nanoparticles. Observable in both scenarios is a discernible alteration in flow patterns across various time frames.

**Figure 20:**
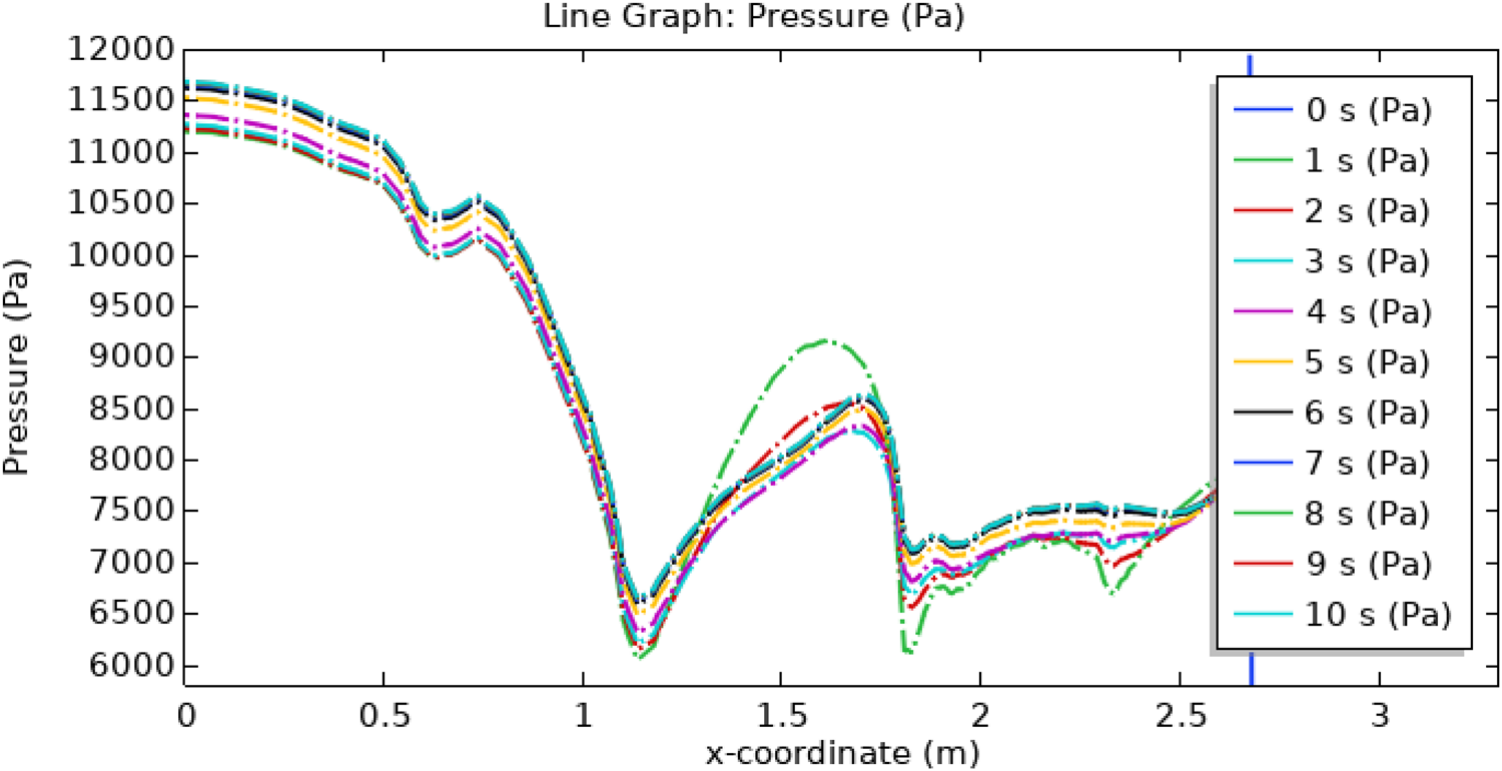
Line graph of blood pressure with magnetized nanoparticles.

**Figure 21:**
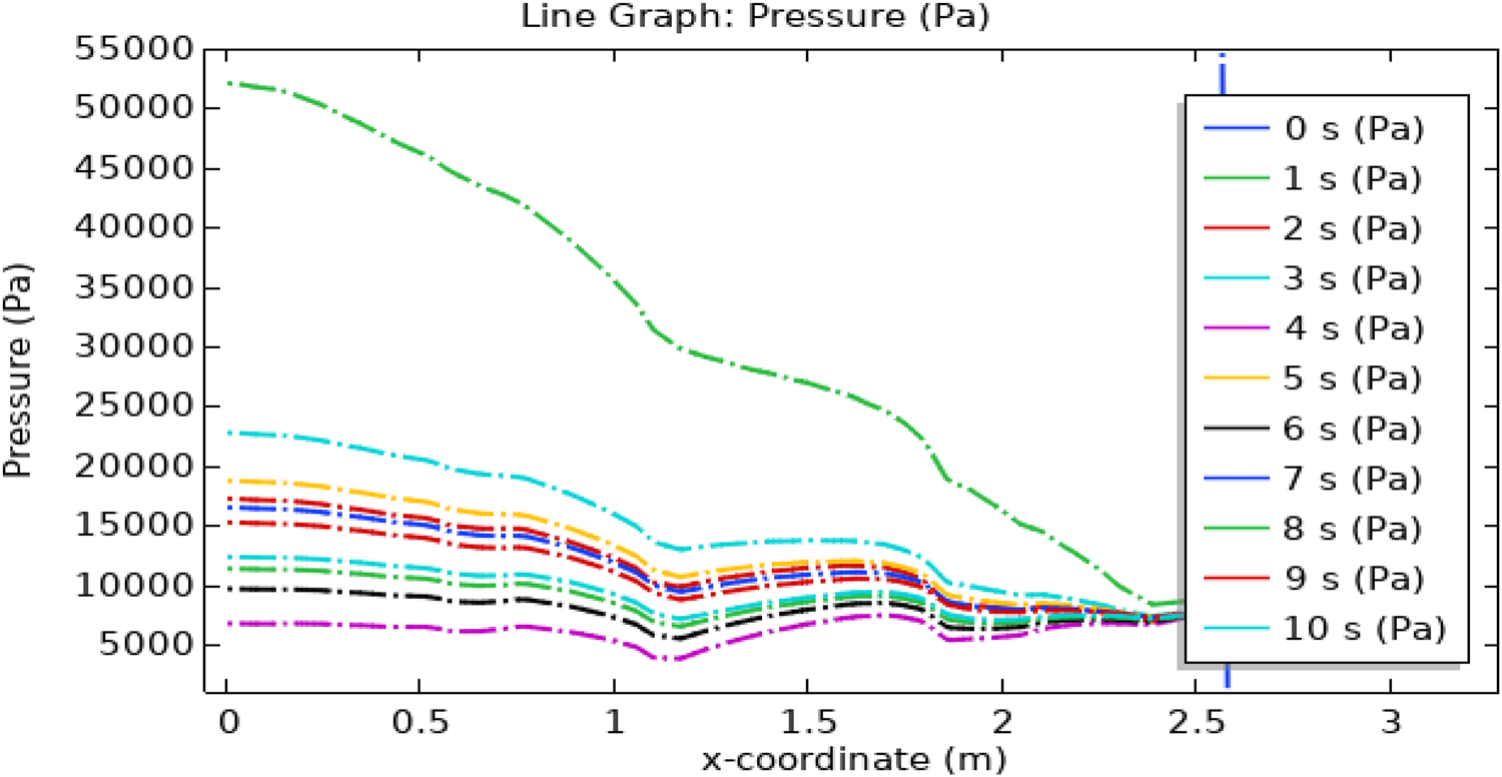
Line graph of blood pressure without magnetized nanoparticles.

In Fig. 20, the depicted arterial pressure profile demonstrates a notable decline from an initial reading of 12,700–6000 pa subsequent to the introduction of hybrid nanoparticles comprising gold and silver in conjunction with the application of a consistent magnetic field. Conversely, as illustrated in Fig. 21, the arterial pressure experiences a rapid surge, reaching approximately 50,000 pa within a 1-s interval. This substantial pressure escalation raises concerns regarding its physiological compatibility with human arteries, as such an intense force has the potential to instigate arterial rupture. As can be seen from the graph the pressure is highest at the inlet of the artery at all times but the highest pressure is for 1 s and the lowest is for 4 s. As the blood flows through the artery passing through triangular stenosis and thrombus of radius 0.1 m the pressure drops to 10,000 pa, and for elliptical stenosis and thrombus of radius 0.05 m, it drops to its minimum value of 6000 pa.

**Figure 22:**
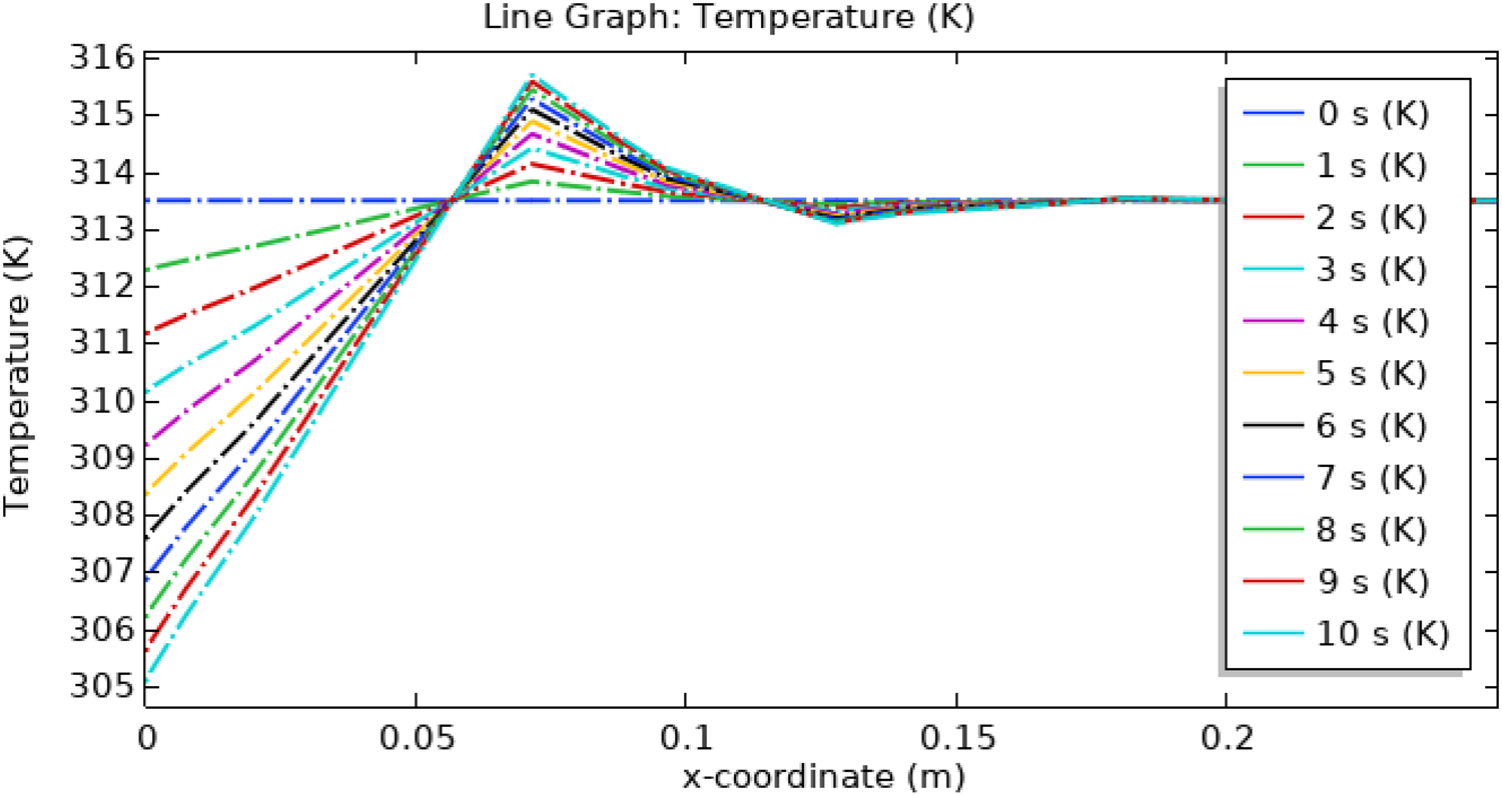
Line graph of blood temperature with magnetized nanoparticles.

**Figure 23:**
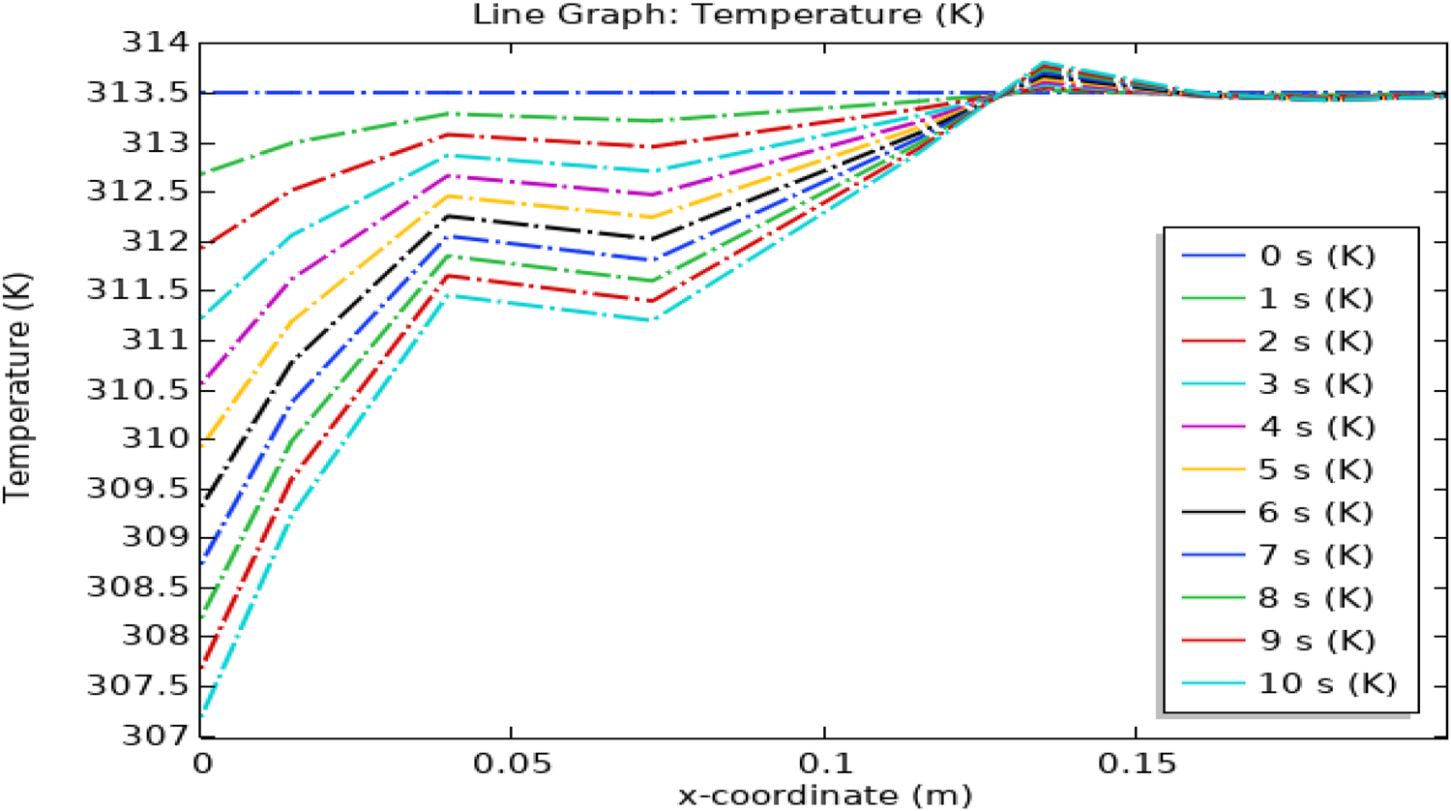
Line graph of blood temperature without magnetized nanoparticles.

Figure 22, shows the temperature profile inside the artery. At the inlet, the temperature is minimum for all times and then it starts increasing to 315.5 K as shown by the color legend then it starts decreasing as the blood flows through some distance after that it remains constant throughout the artery. Temperature also depends on time as shown by legend. Illustrated in Fig. 23, is a comprehensive temperature distribution spanning the entire length of the stenosed artery. This depiction pertains to a scenario wherein magnetized hybrid nanoparticles are absent, and the identical underlying assumptions are upheld for comparative purposes.

## Conclusion

A mathematical and computational model was developed in this work to investigate the characteristics of blood flow inoculating silver and gold hybrid nanoparticles and generated magnetic fields in numerous stenosed arteries with irregular thrombosis. Newtonian characteristics of blood were calculated computationally using the second-order finite element method (FEM). It is imperative to remember that in certain situations, induced magnetic fields can be generated for magnetic medication aiming. The injection of hybrid nanoparticles into the circulation, in conjunction with the application of an external magnetic field, has been studied as a way of improving targeted medication delivery and thermal treatment. A generated magnetic field can direct nanoparticles to specific target areas, increasing their accumulation and therapeutic efficiency. The following are some of the important findings of the current inquiry, as indicated via graphical analysis:

- The laminar flow experiment revealed that blood flow velocity fluctuates all along the model due to erratic thrombus and arterial plaque.
- The induced magnetic field causes an increase in arterial pressure decrease, which is most visible at the ideal flow rate.
- The magnetic field is observed to resist fluid mobility in the center of the artery, but the nanoparticle volume fraction increases motility.
- The induced magnetic increased the impedance to the flow, however, the nanoparticle volume fraction decreased it.
- The increase in the volume percentage of nanoparticles lowers the temperature profile.
- Temperature elevation varied according to the concentration and dispersion of the nanoparticles, as well as the strength and duration of the applied magnetic field.
- Higher nanoparticle concentrations and higher magnetic fields resulted in greater temperature rises.
- The temperature of the fluid decreases as the stenosis form parameter and depth of stenosis rise, resulting in a drop in blood flow in the artery.
- The inclusion of nanoparticles changed the flow patterns and resistance inside the pipes, resulting in pressure drop differences as compared to baseline circumstances.
- The variations in wall shear stress and pressure drop observed revealed the effect of hybrid nanoparticles on the overall hemodynamics of stenosed and thrombosed arteries.
